# Genomic screening reveals ubiquitin-like modifier activating enzyme 1 as a potent and druggable target in c-MYC-high triple negative breast cancer models

**DOI:** 10.1093/pnasnexus/pgac232

**Published:** 2022-10-11

**Authors:** Sheeba Jacob, Tia H Turner, Jinyang Cai, Konstantinos V Floros, Ann K Yu, Colin M Coon, Rishabh Khatri, Mohammad A Alzubi, Charles T Jakubik, Ynes M Bouck, Madhavi Puchalapalli, Mayuri Shende, Mikhail G Dozmorov, Sosipatros A Boikos, Bin Hu, J Chuck Harrell, Cyril H Benes, Jennifer E Koblinski, Carlotta Costa, Anthony C Faber

**Affiliations:** Department of Oral and Craniofacial Molecular Biology, Philips Institute for Oral Health Research, VCU School of Dentistry and Massey Cancer Center, Virginia Commonwealth University, Richmond, VA 23298, USA; Department of Pathology, Virginia Commonwealth University School of Medicine, Richmond, VA 23298, USA; Wright Center for Clinical and Translational Research, Virginia Commonwealth University School of Medicine, Richmond, VA 23298, USA; Department of Oral and Craniofacial Molecular Biology, Philips Institute for Oral Health Research, VCU School of Dentistry and Massey Cancer Center, Virginia Commonwealth University, Richmond, VA 23298, USA; Department of Oral and Craniofacial Molecular Biology, Philips Institute for Oral Health Research, VCU School of Dentistry and Massey Cancer Center, Virginia Commonwealth University, Richmond, VA 23298, USA; Department of Oral and Craniofacial Molecular Biology, Philips Institute for Oral Health Research, VCU School of Dentistry and Massey Cancer Center, Virginia Commonwealth University, Richmond, VA 23298, USA; Department of Oral and Craniofacial Molecular Biology, Philips Institute for Oral Health Research, VCU School of Dentistry and Massey Cancer Center, Virginia Commonwealth University, Richmond, VA 23298, USA; Department of Oral and Craniofacial Molecular Biology, Philips Institute for Oral Health Research, VCU School of Dentistry and Massey Cancer Center, Virginia Commonwealth University, Richmond, VA 23298, USA; Department of Pathology, Virginia Commonwealth University School of Medicine, Richmond, VA 23298, USA; Integrative Life Sciences Program, Virginia Commonwealth University, Richmond, VA 23298, USA; Center for Cancer Research, Massachusetts General Hospital Cancer Center and Department of Medicine, Harvard Medical School, Boston, MA 02129, USA; Department of Oral and Craniofacial Molecular Biology, Philips Institute for Oral Health Research, VCU School of Dentistry and Massey Cancer Center, Virginia Commonwealth University, Richmond, VA 23298, USA; Department of Pathology, Virginia Commonwealth University School of Medicine, Richmond, VA 23298, USA; Department of Pathology, Virginia Commonwealth University School of Medicine, Richmond, VA 23298, USA; Department of Biostatistics, Virginia Commonwealth University, Richmond, VA 23298, USA; Hematology, Oncology and Palliative Care, School of Medicine and Massey Cancer Center, Virginia Commonwealth University, Richmond, VA 23298, USA; Department of Pathology, Virginia Commonwealth University School of Medicine, Richmond, VA 23298, USA; Department of Pathology, Virginia Commonwealth University School of Medicine, Richmond, VA 23298, USA; Wright Center for Clinical and Translational Research, Virginia Commonwealth University School of Medicine, Richmond, VA 23298, USA; Integrative Life Sciences Program, Virginia Commonwealth University, Richmond, VA 23298, USA; Center for Cancer Research, Massachusetts General Hospital Cancer Center and Department of Medicine, Harvard Medical School, Boston, MA 02129, USA; Department of Pathology, Virginia Commonwealth University School of Medicine, Richmond, VA 23298, USA; Center for Cancer Research, Massachusetts General Hospital Cancer Center and Department of Medicine, Harvard Medical School, Boston, MA 02129, USA; Department of Oral and Craniofacial Molecular Biology, Philips Institute for Oral Health Research, VCU School of Dentistry and Massey Cancer Center, Virginia Commonwealth University, Richmond, VA 23298, USA

**Keywords:** UBA1, ER stress, CRISPR, targeted therapies, c-MYC

## Abstract

Triple negative breast cancer (TNBC) accounts for over 30% of all breast cancer (BC)-related deaths, despite accounting for only 10% to 15% of total BC cases. Targeted therapy development has largely stalled in TNBC, underlined by a lack of traditionally druggable addictions like receptor tyrosine kinases (RTKs). Here, through full genome CRISPR/Cas9 screening of TNBC models, we have uncovered the sensitivity of TNBCs to the depletion of the ubiquitin-like modifier activating enzyme 1 (UBA1). Targeting UBA1 with the first-in-class UBA1 inhibitor TAK-243 induced unresolvable endoplasmic reticulum (ER)-stress and activating transcription factor 4 (ATF4)-mediated upregulation of proapoptotic NOXA, leading to cell death. c-MYC expression correlates with TAK-243 sensitivity and cooperates with TAK-243 to induce a stress response and cell death. Importantly, there was an order of magnitude greater sensitivity of TNBC lines to TAK-243 compared to normal tissue-derived cells. In five patient derived xenograft models (PDXs) of TNBC, TAK-243 therapy led to tumor inhibition or frank tumor regression. Moreover, in an intracardiac metastatic model of TNBC, TAK-243 markedly reduced metastatic burden, indicating UBA1 is a potential new target in TNBC expressing high levels of c-MYC.

Significance Statement Genomic screening of TNBC cell lines revealed broad sensitivity to depletion of the E1 ubiquitin enzyme, UBA1. Disrupting UBA1 with the first-in-class inhibitor TAK-243 in TNBC models induces ER-stress through an ATF4-NOXA axis that is dependent on c-MYC, leading to apoptosis, both in vitro and in vivo, tumor growth, and metastatic inhibition.

## Introduction

Triple negative breast cancer (TNBC) accounts for up to 15% of breast cancers (BCs) and is often lethal, in particular to young, African-American women (1). TNBC are extremely heterogeneous in terms of genomic alterations (2).

The implementation of targeted therapies has been revolutionary in different cancer paradigms including BC. Estrogen receptor (ER) inhibitors and human epidermal growth factor receptor 2 (HER2) inhibitors have both been integrated into BC patient care, where nearly 90% of patients positive for one of these alterations now survive (5). TNBCs by definition are not positive for hormone receptors or excessive HER2 receptors. Reoccurring alterations are mostly limited to loss of phosphatase and tensin homolog (PTEN) (∼ 35% of cases), PIK3CA activating mutations (∼ 9% of cases) and loss of breast cancer (BRCA) genes (19.5% of cases) (2). Even so, treatment with phosphoinositide 3-kinase (PI3K) inhibitors has not been successful in TNBC, and poly (ADP-ribose) polymerase (PARP) inhibitor therapy has not been as successful in BRCA mutant TNBC (6) as it has in BRCA mutant ovarian cancer (7). Extreme heterogeneity may underlie some of the lack of consistency in response to both of these classes of inhibitors (8, 9). Despite this, TNBCs have many common characteristics for instance, about 80% are derived from basal-like tissue TNBCs in general possess strong immunosuppressive qualities including high programmed death-ligand 1 (PD-L1) expression (10) and TNBCs have a tendency to strike younger African-American women, where it is largely more aggressive than other BCs (11). Altogether, the genomic and clinical evidence suggest that implementation of targeted therapies will likely require an expansion of potential targets.

## Results

### Genomic screening of TNBC reveals hits in the ubiquitin–proteasome system (UPS)

**Fig. 1.**
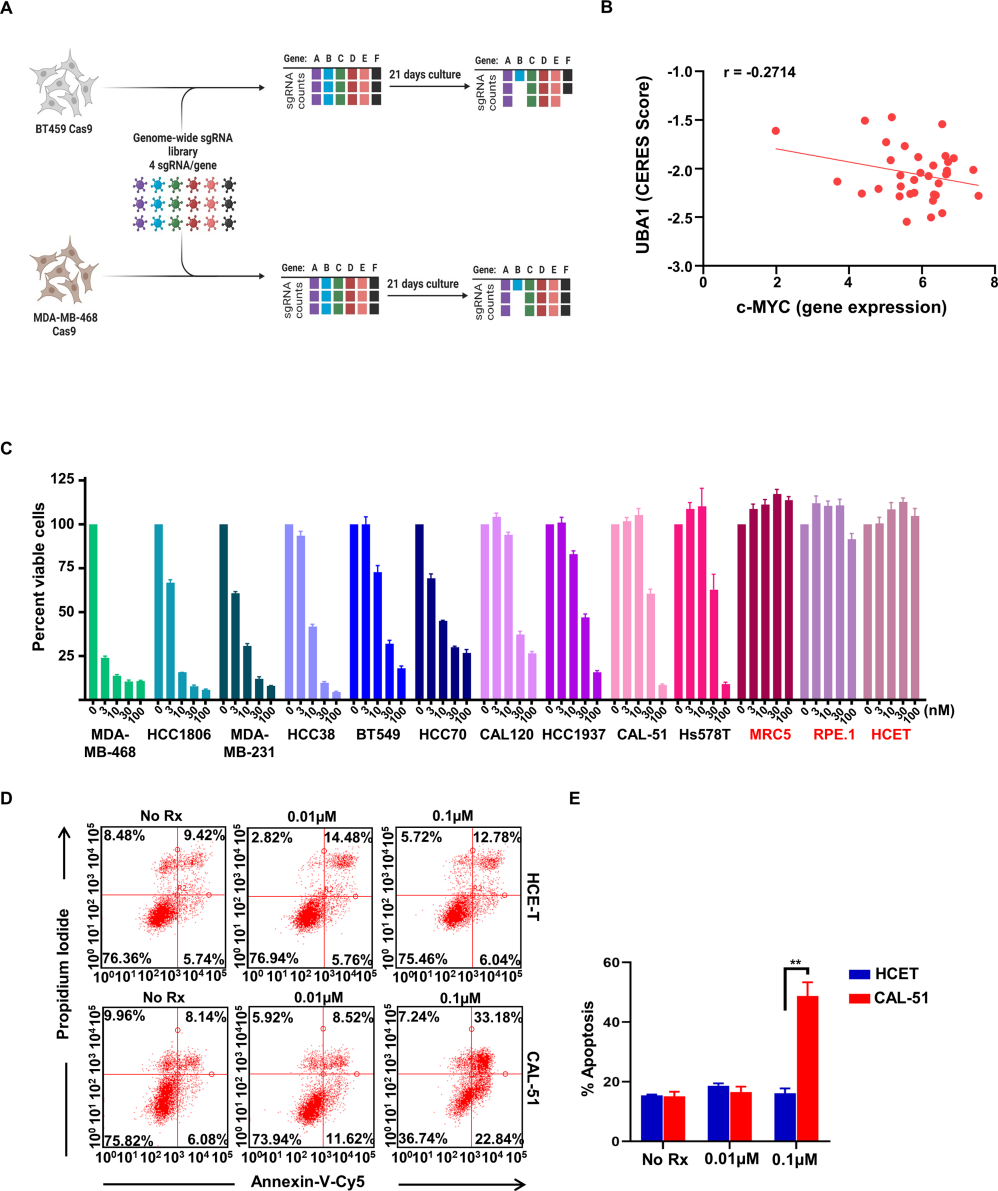
UBA1 is a target in TNBC. (A) Schema of the TNBC genetic screen. Whole genome of the TNBC cell lines (BT-549 and MDA-MB-468) was followed by UBA1 depletion in nine TNBC cell lines. Created with BioRender.com (B) Graph shows a strong negative correlation (*r* = −0.2714) between the CERES score from targeting the UBA1 gene in CRISPR knock-out cells and c-MYC expression obtained in various BC models (C) Graph represents % of viable cells assessed by CellTiter-Glo in TNBCs and normal tissue-derived MRC5, human retinal pigment epithelial-1 (RPE-1), and human corneal epithelial cell-transfrmed (HCE-T) cells (red) following 72-h treatment with TAK-243 at the indicated concentration. (D) Flow cytometric analysis showing annexin-V-Cy5 and propidium iodide staining in HCE-T and CAL-51 cells following 24-h treatment with TAK-243 at the indicated concentration. (E) The graph represents % of apoptosis assessed by flow cytometry in HCE-T and CAL-51 cells following 24-h treatment with TAK-243 at the indicated concentration. Error bars are SEM *n* = 3 and **P < 0.01.

In order to capture the full scope of potential targets, we performed whole genome CRISPR/Cas9 screening, covering approximately 20,000 target genes with four single-guide (sg)RNAs targeting each gene, in two TNBC cell lines: BT-549 (derived from primary breast tumor) and MDA-MB-468 (derived from pleural effusion) (Fig. 1A). Following 21 d of selection, the % of surviving cells with each sgRNA was calculated by Negative Binomial Distrubtion (STARS) analyses (12) (top hits are listed in Supplementary Material Table S1). Interestingly, the analyses uncovered several gene hits in the UPS including multiple ubiquitin-specific peptidases (USPs) and proteasome subunits, as well as previously reported TNBC survival genes like MYC (Supplementary Material Tables S1 and S2) (13, 14). Among the highest-ranked genes in both cell lines was Ubiquitin-Like Modifier Activating Enzyme 1 (UBA1) (also known as UBE1) (Supplementary Material Tables S1 and S2). The Dependency Map (DepMap) portal also revealed UBA1 and other ubiquitin-related genes (Supplementary Material Table S3) as essential genes based on their genomic copy number (CN) adjusted essentiality score (CERES score) scores (lower the CERES score, higher is the essentiality of the gene) in TNBC CRISPR knock-out cell lines. Furthermore, we compared the CERES scores of UBA1 to the expression level of c-MYC across different BC models, which revealed a strong negative correlation (Fig. 1B and Supplementary Material Fig. S1A) and found UBA1 targeting was in general more toxic in c-MYC high cells, which clustered in the basal-like molecular subset, where most TNBC derive from(15). UBA1 is the E1 ubiquitin-activating enzyme and represents a critical node of control in the UPS (16, 17). Based on the sensitivity of both cell lines to UBA1 depletion, we expanded our evaluation to an additional 7 TNBC cell lines (HCC1806, HCC70, SUM149PT, MDA-MB-231, Hs578T, MDA-MB-436, and HCC1937), using 10 UBA1-targeting sgRNAs. All nine cell lines demonstrated sensitivity to depletion of UBA1 (Supplementary Material Fig. S1B), suggesting broad efficacy throughout diverse TNBC models. TAK-243 is a novel UBA1 inhibitor (17–19) currently in clinical evaluation. To assess the potential of TAK-243 in recapitulating the genetic screens results, 14 TNBC cell lines and three normal tissue-derived cell lines of different origins were treated with TAK-243. We found a striking difference between the sensitivity of the TNBC cell lines and normal tissue-derived cells, suggesting that TAK-243 has the potential to specifically target TNBC cells (Fig. 1C and Supplementary Material Fig. S1C).

### TAK-243 induces unresolvable ER stress, mediated by activating transcription factor 4 (ATF4) NOXA

**Fig. 2.**
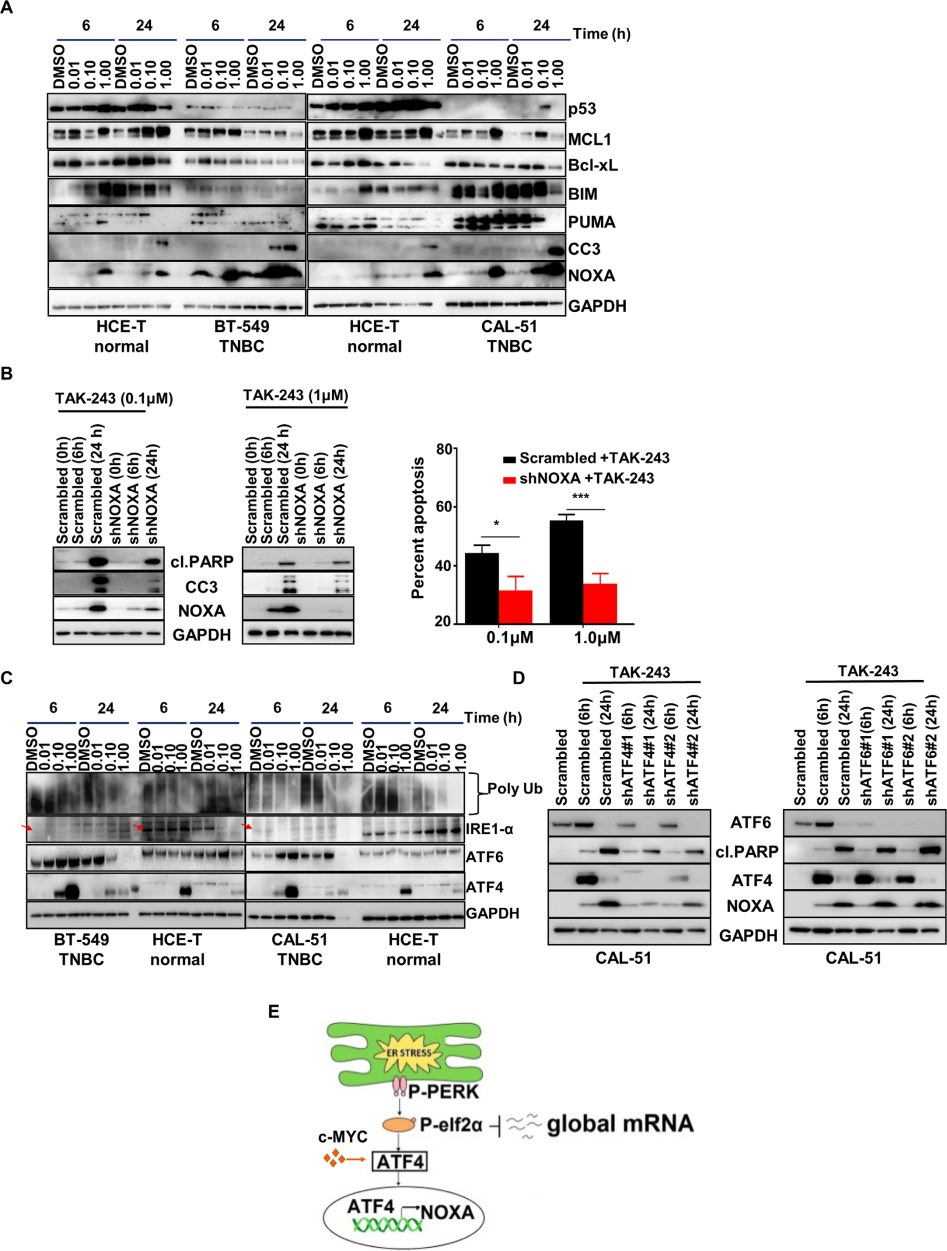
TAK-243 induces ER stress in TNBC. (A) Western blot analysis showing the dose response and time course of the effects of TAK-243 on apoptosis in TNBC (CAL-51 and BT-549) and normal tissue-derived HCE-T cells, as assessed by immunoblotting for p53, MCL-1, Bcl-xL, BIM, PUMA, cleaved Caspase 3 (CC3) and NOXA. Glyceraldehyde-3-phosphate dehydrogenase (GAPDH) was used as a loading control. (B) Western blot analysis showing stable knockdown of NOXA. Reduced NOXA protects from TAK-243 toxicity in CAL-51 cells as assessed by immunoblotting for cleaved PARP, CC3 and NOXA. GAPDH was used as a loading control (left panel). The right panel indicates % apoptosis assessed by flow cytometry in pLKO.1-shRNA control and CAL-51 NOXA knockdown stable cells treated with TAK-243 at the indicated concentration. (C) Western blot analysis showing the dose response and time course of the effects of TAK-243 on UPR proteins in TNBC (CAL-51 and BT-549) and normal tissue-derived HCE-T cells, as assessed by immunoblotting for ATF4 and ATF6. Polyubiquitin (polyUb) indicates ubiquitin engagement in these cells. GAPDH was used as a loading control. (D) Western blot analysis demonstrating the role of ATF4 in the regulation of NOXA as assessed by immunoblotting of scrambled or shATF4/shATF6 transduced CAL51 cells. (E) Model for TAK-243 efficacy in TNBC. For (B), error bars are SEM, *n* = 3 and *P < 0.05; ***P < 0.001.

Encouraged by the sensitivity to TAK-243 in the viability assays (Fig. 1C), we investigated whether inhibiting UBA1 was sufficient to induce cell death. Indeed, TAK-243 induced apoptotic cell death in TNBCs but had very little effect in normal tissue-derived human corneal epithelial cell-transformed (HCE-T) cells (Fig. 1D and E). B-cell lymphoma 2 (BCL-2) family proteins mediate apoptosis (20), and examination of the level of these proteins demonstrated that TAK-243 did not downregulate key antiapoptotic proteins in TNBC, nor did consistently upregulate the proapoptotic protein BCL-2 interacting mediator (BIM). Interestingly, NOXA, another proapoptotic protein, was strongly induced in the TNBC models (12.5-fold in BT-549 and 18.4-fold in CAL-51) but less so in HCE-T cells (2-fold) treated with 1µM TAK-243 for 24h (Fig. 2A and Supplementary Material Fig. S2A).

To test whether NOXA was necessary for the observed effect of TAK-243, shRNA mediated depletion of NOXA was employed (Fig. 2B left panel). NOXA loss in TNBC cells led to mitigation of TAK-243-induced cell death (Fig. 2B, right panel and Supplementary Material Fig. S2B), demonstrating a critical role for NOXA in TAK-243 efficacy. NOXA is activated by both p53 and ER-stress, and disruption of protein homeostasis often induces ER-stress and the unfolded protein response (UPR), as well as the p53 response (21). To investigate the mechanism of NOXA upregulation, we evaluated both p53 response (Fig. 2A and Supplementary Material Fig. S2A) and ER stress response (Fig. 2C and Supplementary Material Fig. S2C). TAK-243 did not markedly induce p53 in neither the p53 mutant BT-549, MDA-MB-468, and HCC70 cells nor the p53 wild-type CAL-51 cells (Fig. 2A and Supplementary Material Fig. S2A). The UPR consists of three main pathway arms initiated by ER stress: (1) Protein kinase RNA-like reticulum kinase (PERK), which activates eukaryotic initiation factor 2 (eIF2 alpha) to repress general translation and promote specific translation of a set of mRNAs including ATF4; (2) Inositol-requiring enzyme 1 (IRE1); and (3) ATF6 (22). Interestingly, we found TAK-243 strongly induced ATF4 expression in every TNBC model analyzed, and to a lesser degree also ATF6 expression, although it is variable across models (Fig. 2C and Supplementary Material Fig. S2C). The ER stress response induced by TAK-243 in the normal tissue-derived cells was dampened relative to the response in TNBC cells with less marked ATF4 and NOXA upregulation following TAK-243 exposure (Fig. 2C), suggestive of a tempered ER stress response correlating with decreased toxicity in the normal tissue-derived cells. To probe the potential functional role of ATF4 and ATF6 in NOXA upregulation, and response to TAK-243, ATF4, and ATF6 expression were reduced using specific shRNAs. While ATF6 knockdown did not impact NOXA induction or cell death, ATF4 depletion mitigated both NOXA upregulation and cell death following TAK-243 treatment (Fig. 2D). These data support a model where blocking UBA1 induces the UPR, leading to unresolvable ER-stress, ATF4-mediated NOXA upregulation, and apoptosis in TNBC (Fig. 2E).

### TAK-243 induces some tumor regressions in TNBC patient-derived xenografts

**Fig. 3.**
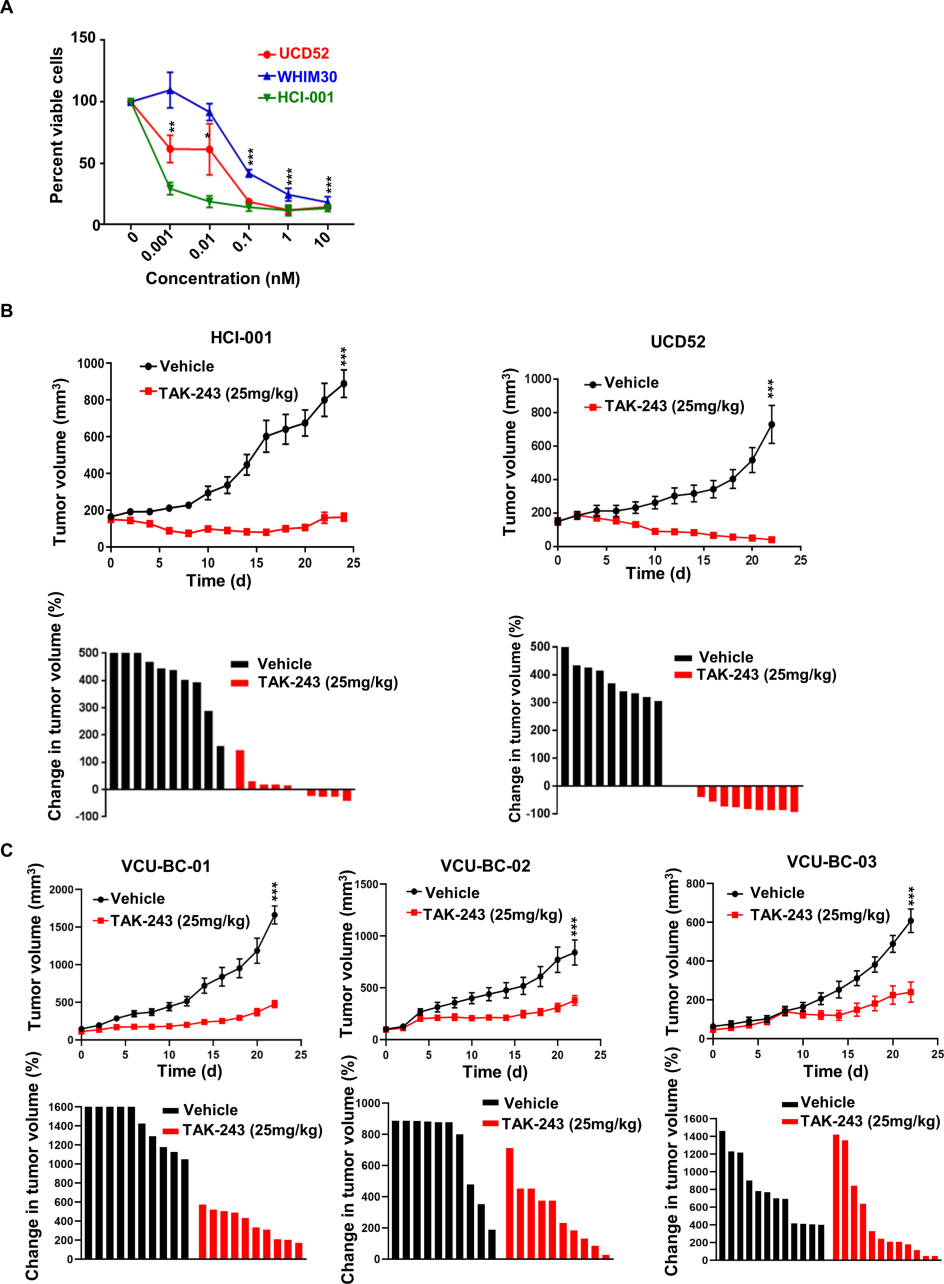
TAK-243 is highly effective in inhibiting TNBC tumor growth. (A) Graph represents % viable cells in ex vivo TNBC cell cultures (WHIM30, UCD52, and HCI-001) following  72-h treatment with TAK-243 at the indicated concentrations. (B) Antitumor activity of TAK-243 was assessed by tumor volume over time in mice bearing patient-derived TNBC xenografts HCI-001, UCD52, and (C) TNBC PDXs developed at VCU- VCU-BC-01, VCU-BC-02, and VCU-BC-03. Waterfall plot represents the change in tumor volume % of each tumor from their initial tumor size. For (A), error bars are SEM and * *P* < 0.05, **P < 0.01, ***P < 0.001 and (B) *** treatment effect significance < 0.01, linear mixed model analysis.

We recently characterized several PDX models and corresponding cell lines (23); evaluation of TAK-243 in three PDX spheroid cell cultures demonstrated similar TAK-243 efficacy (Fig. 3A) as we found in the TNBC cell lines (Fig. 1C). Encouraged by the broad, albeit variable, sensitivity of TAK-243 across these TNBC PDX spheroid models, we treated female NOD scid gamma (NSG) mice bearing these TNBC PDX tumors and additional TNBC PDX models developed at Virginia Commonwealth University (VCU) with TAK-243 (Supplementary Material Table S4) (18). Treatment was sufficient to induce strong responses with overt tumor regression in one PDX model and with strong, albeit a more mixed response, in the other four PDX models (Fig. 3B and C). Immunoblot analysis of control and TAK-243 treated tumor tissues (HCI-001, VCU-BC-01, and VCU-BC-03) confirmed pharmacodynamic (PD) modulation by polyubiquitnation inhibition (Supplementary Material Fig. S3A). Immunohistochemical analysis of the expression of the cell death marker cl. Caspase 3 (CC3) further validated our findings of increased cell death following TAK-243 treatment compared to vehicle-treated PDX mice (Supplementary Material Fig. S3B). Treatment tolerability was indicated by weight maintenance throughout the study (Supplementary Material Fig. S4). Overall, these data are consistent with the in vitro findings from the TNBC cell lines, demonstrating a high degree of activity across TNBC models through targeting of UBA1, which can be recapitulated with the small molecule UBA1 inhibitor, TAK-243.

### TAK-243 decreases primary metastases in a metastatic mouse model of TNBC

**Fig. 4.**
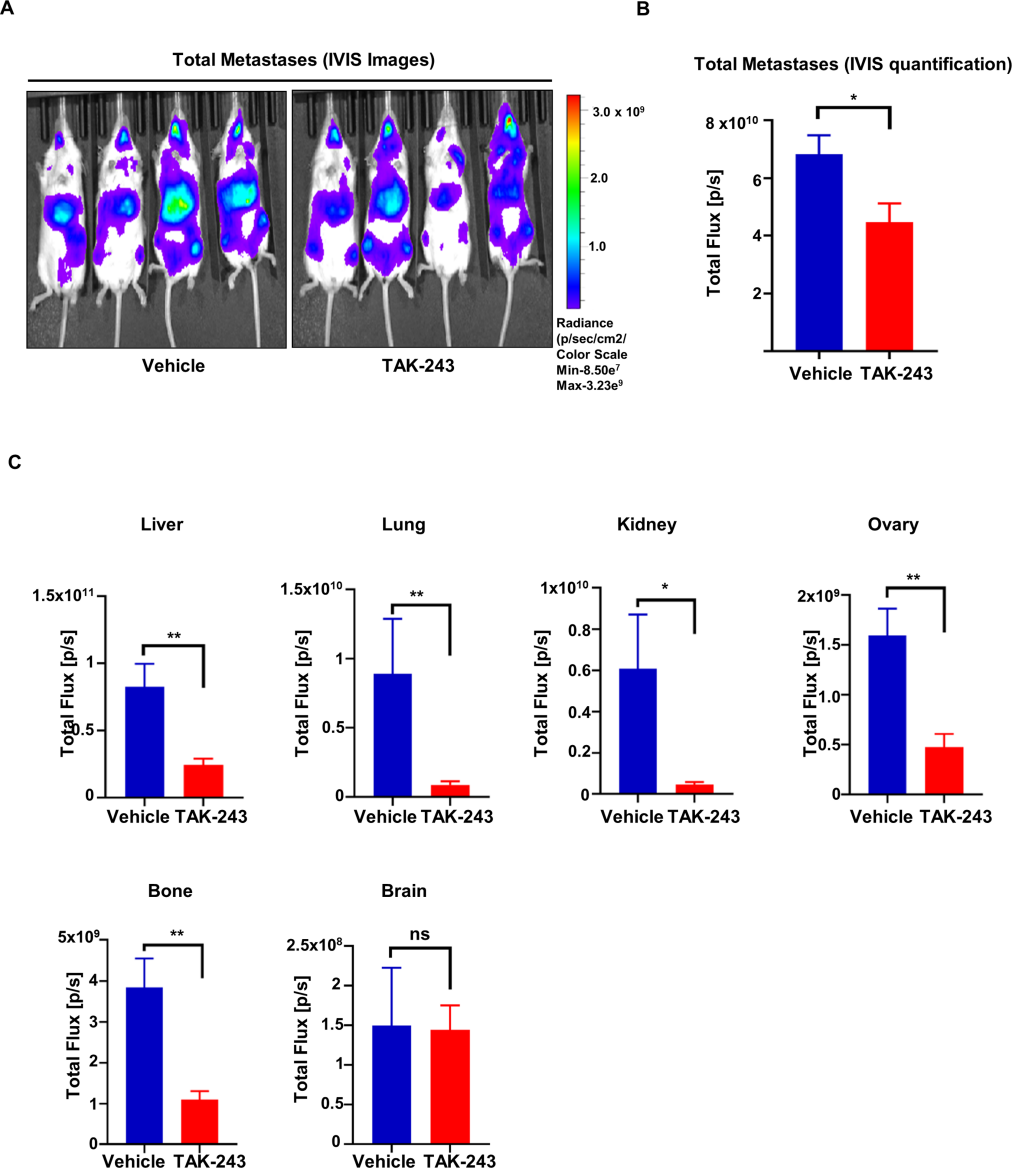
TAK-243 decreases metastases in TNBC. (A) Total metastases were imaged in vivo in the MDA-MB-231 tomato–luciferase model. (B) Graph represents total metastases quantified in vivo in the MDA-MB-231 tomato–luciferase model. (C) Graph represents metastases quantified ex vivo in the organs (liver, lungs, kidney, ovaries, bone, and brain) of vehicle- and TAK-243-treated mice (*P < 0.05; **P < 0.01, ns: not significant).

Metastasis is responsible for almost all TNBC deaths. TNBC metastasizes to the lymph nodes, liver, lungs, bone, and brain. We evaluated the effect of TAK-243 on metastatic growth using an intracardiac MDA-MB-231-tomato-luciferase injection model. Drug treatment did not begin until 10 d after the injection of the cells allowing time for the cells to extravasate to all organs and begin to grow. Importantly, we observed an overall decrease in the metastatic burden of the NSG mice treated with TAK-243 [(25 mg/kg), Fig. 4A and B] and in primary metastatic regions such as the lung, liver, and bones as well as the ovaries, and kidney (Fig. 4C and Supplementary Material Fig. S5). The luciferase signals emitted from different organs were more appreciable than the luciferase signals emitted from the whole body due to skin pigments and hair that can significantly scatter light from underlying organs (24, 25). These data demonstrate that TAK-243 decreases TNBC metastatic burden. A decrease in the brain metastases was not observed, which may be due to the short treatment interval of TAK-243 that may be insufficient to inhibit brain metastases as opposed to the inhibition reported by Liu et al. in their study on glioblastoma cells (26).

**Fig. 5.**
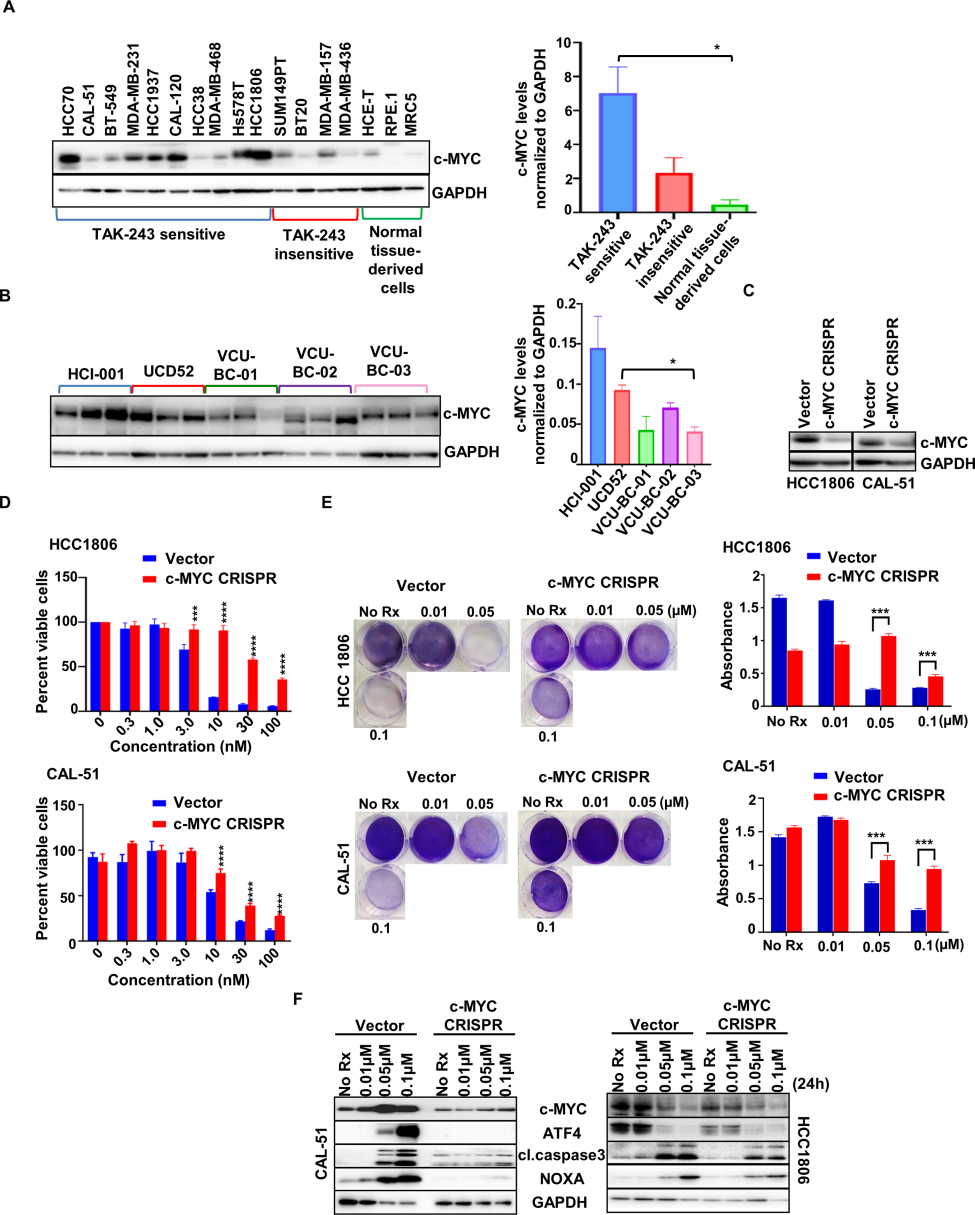
c-MYC mediates sensitivity to TAK-243 in TNBC. (A) Western blot analysis showing basal levels of c-MYC in TNBC cell models sensitive to TAK-243, TNBC cell models insensitive to TAK-243, and normal tissue-derived cell lines (left panel). The right panel indicates c-MYC levels normalized to GAPDH in the TAK-243 sensitive TNBC cell models, TAK-243 insensitive TNBC cell models and normal tissue-derived cell lines (* *P* < 0.05; used Bonferroni correction). (B) Immunoblot showing c-MYC expression in PDX tumor lysates. The right panel indicates c-MYC levels normalized to GAPDH in the PDX tumor lysates (*P < 0.05; used Bonferroni correction). (C) Immunoblot showing c-MYC expression in pLenti CRISPR v2 (control) and pLenti CRISPR c-MYC stable lines. (D) Graph represents % viable cells assessed by Cell Titer-Glo in two pLenti CRISPR v2 (control, blue) and pLenti CRISPR c-MYC stable lines (red) following  72-h treatment with TAK-243 at the indicated concentrations. (E) Crystal violet assay in pLenti CRISPR v2 (vector) and pLenti CRISPR c-MYC stable lines treated for 4 d with TAK-243 at the indicated concentrations. Graph (right panel) represents absorbance measured at 570 nm of the crystal violet assay. (F) Immunoblot showing expression of cl.PARP, CC3, and NOXA in pLenti CRISPR v2 (vector) and pLenti CRISPR c-MYC stable cells treated with TAK-243 for 24-h at the indicated concentrations. For (D) and (E), error bars are SEM and *** *P* < 0.001; ****P < 0.0001.

**Fig. 6.**
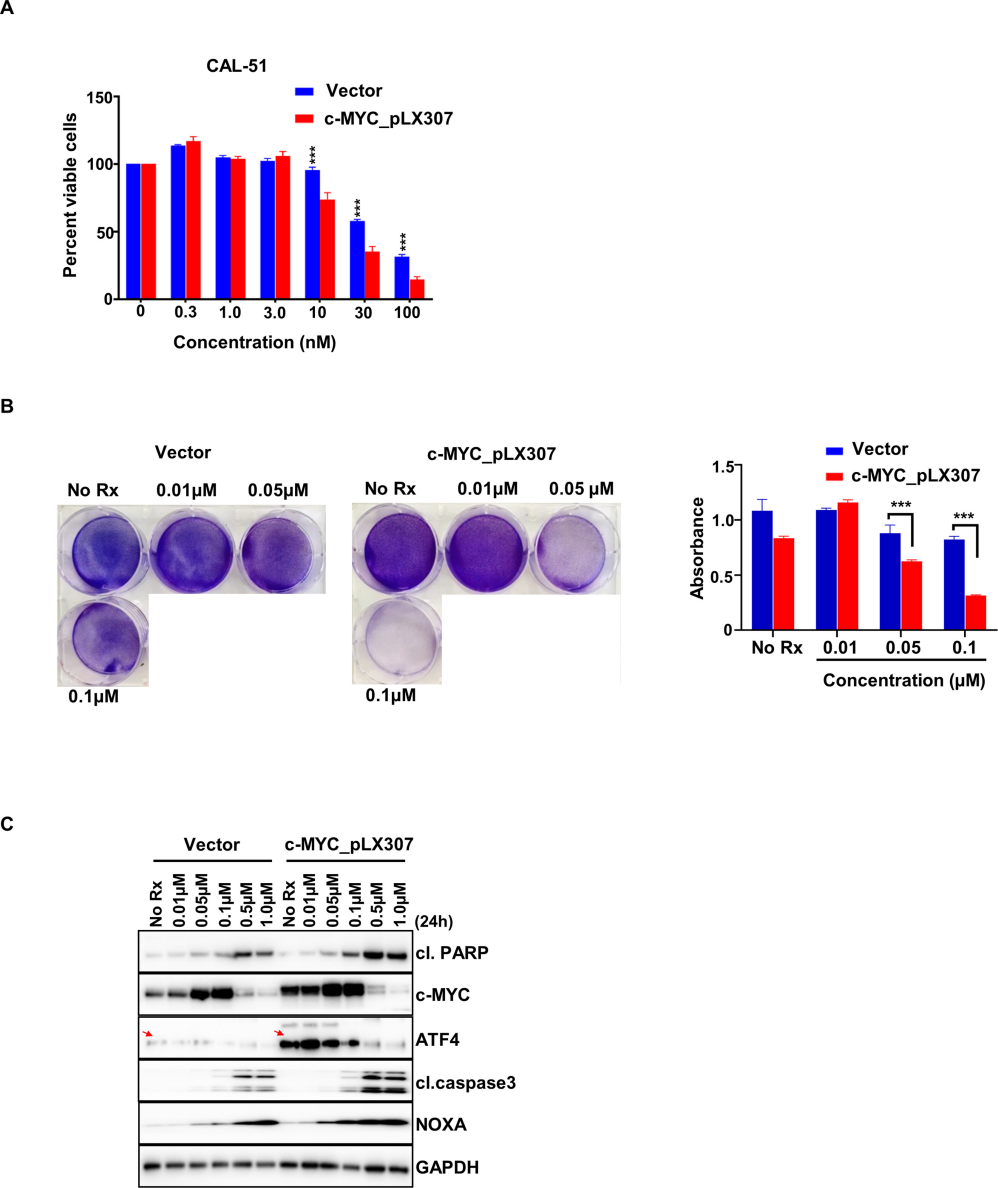
c-MYC overexpression increases sensitivity to TAK-243 in TNBC. (A) Graph represents % viable cells assessed by Cell Titer-Glo in lentiviral pLX307 (vector) and c-MYC_pLX307 expressing CAL-51 cells following  72-h treatment with TAK-243 at the indicated concentrations. (B) Crystal violet assay in lentiviral pLX307 (vector) and c-MYC_pLX307 expressing CAL-51 cells treated for 3 d with TAK-243 at the indicated concentrations. (C) Immunoblot showing expression of cl. PARP, CC3, and NOXA in lentiviral pLX307 (vector) and c-MYC_pLX307 expressing CAL-51 treated with TAK-243 for  24-h at the indicated concentrations.

### c-MYC is a functional biomarker for TAK-243 activity across TNBC models

As high c-MYC levels are detected often in TNBC (27) and we recently demonstrated that n-MYC, a closely related paralog of c-MYC, sensitizes cancer cells to NOXA-mediated toxicity (28), we asked whether c-MYC expression could be a potential factor in TAK-243 efficacy in TNBC. To do this, we analyzed c-MYC expression across our panel of TNBC cell lines and normal tissue-derived cells. We found an increase in c-MYC expression between the TAK-243 sensitive TNBCs and the normal tissue-derived cells (Fig. 5A, left and right panels). Moreover, we detected increased expression of c-MYC in the TAK-243 sensitive TNBCs compared to insensitive TNBCs (Fig. 5A, left and right panels). We also noted that in the PDXs, the best responders to TAK-243 (HCI-001 and UCD52) had higher levels of c-MYC compared to the other PDXs tested (VCU-BC-01, VCU-BC-02, and VCU-BC-03) (Fig. 5B), suggesting that c-MYC expression might correlate with sensitivity to TAK-243 in in vivo as well.

To determine if c-MYC can modulate TAK-243 sensitivity, we reduced c-MYC expression in HCC1806 and CAL-51 cells using CRISPR/Cas9 technology (Fig. 5C). Indeed, downregulation of c-MYC expression decreased the sensitivity of TNBC cells to TAK-243 (Fig. 5D and E). Consistent with the ability of MYC to induce an ER-stress response (29) and ATF4 expression (30–32), cells with reduced c-MYC expression had decreased induction of ATF4 and NOXA following TAK-243 treatment. Consistent with these findings, there was a reduction in TAK-243-mediated cell death in the MYC-reduced cells, as determined by CC3 expression (Fig. 5F). To confirm the involvement of c-MYC in TAK-243 sensitivity in TNBC, we overexpressed c-MYC with lentiviral producing c-MYC-expressing plasmids. Consistent with the CRISPR experiment results, exogenous c-MYC expression in CAL-51 lines increased the sensitivity to TAK-243 even further (Fig. 6A and B). Western blot analyses revealed that c-MYC increased levels of ATF4 and NOXA (Fig. 6C); consistent with this observation, cell death markers were increased in the c-MYC overexpressing cells following TAK-243 (Fig. 6C). Altogether, these data suggest that c-MYC by activating the ATF4/NOXA axis can mediate TAK-243-induced stress response and cell death, and therefore, c-MYC may serve as a functional biomarker for response to TAK-243 in TNBC.

## Discussion

TNBC continues to be routinely treated with chemotherapy, where responses are variable. Due to its varied genomic alteration and fewer druggable targets, TNBC remains one of the most aggressive BC subtype, despite occurring in younger women.

Full genome CRISPR/Cas9 screening offers tangible advantages to past screening efforts by revealing a greater number of phenotypes through improved penetrance, which is a characteristic of genome editing itself compared to, for instance, screening with short-interfering (si) RNA or short-hairpin (sh) RNA libraries (14). We hypothesized that this technique could reveal a drug target that we could capitalize on, given the growing number of clinically available targeted therapies emerging that are designed to interfere with genes outside the traditional kinome.

Cellular protein homeostasis is maintained through a careful balance of protein synthesis and degradation. The consequence of disruptions in this homeostasis causes diverse human diseases, including cancer (18, 19). Protein synthesis is mainly countered in mammalian cells by the UPS, where the protein ubiquitin “tags” proteins for degradation in several cellular proteasomes (19). Here, we found that blocking UBA1, an enzyme responsible for adenylating and capturing a ubiquitin molecule to initiate ubiquitination of cellular proteins (33), was highly toxic to various TNBC models and led to ER-stress and NOXA upregulation, initiating apoptosis.

While proteasome inhibitors have long been investigated as anticancer agents (34), UBA1 inhibitors have taken longer to be developed. Schimmer and colleagues (33) demonstrated that UBA1 was a bona fide drug target in several hematological cancers several years ago. This hypothesis became testable in hematological cancers with the recent discovery and characterization of TAK-243, a specific and potent UBA1 inhibitor (18). Indeed, the same group found that acute myeloid leukemia (AML) models are significantly sensitive to TAK-243 (17, 35), which has also led to its phase 1 trial where TAK-243 will be tested for treating patients with relapsed or refractory AML and chronic myelogenous leukemia (CML) (NCT03816319).

Here, we demonstrate that TAK-243 also has antitumor activity in solid tumors. Indeed, we found that most TNBCs were sensitive to TAK-243, while normal tissue-derived cells were relatively refractory to TAK-243, being roughly an order of magnitude less sensitive than the TNBC cell lines. TAK-243 was sufficient to uniformly block tumor growth in different TNBC PDXs, including the ones developed at VCU (Fig. 3B). Additionally, TAK-243 decreased the metastatic burden of MDA-MB-231-tomato-luc cells in different organs (Fig. 4), demonstrating TAK-243 can decrease both the primary tumor and tumors at various metastatic sites.

Moreover, the oncogenic transcription factor, c-MYC, correlates with TAK-243 sensitivity and has a causative role in mediating TAK-243 sensitivity. c-MYC is an important oncogenic transcription factor in several cancers including TNBC (36, 37). In TNBC, c-MYC contributes to chemotherapy resistance (38). As such, c-MYC is a potential drug target in TNBCs and other cancers but has been historically difficult to target directly due to its protein structure and other characteristics (39). One alternative strategy is to target processes or druggable proteins that are preferentially active in c-MYC expressing TNBCs. For instance, PIM1 expression is higher in c-MYC-expressing TNBCs (40), as is fatty acid oxidation (41) and targeting both these processes has proven to be effective in preclinical models. PIM inhibitor AZD1208 has been tested clinically (e.g. NCT01588548), but the lack of objective responses across hematological and solid tumors has hindered its development (42).

In addition to its roles in supporting proliferation and growth, c-MYC increases ER stress (29, 43) and in particular has an intimate relationship with ATF4 (30). Indeed, both c-MYC and n-MYC have been demonstrated to upregulate ATF4, which may involve the kinase general control nonderepressible 2 (GCN2) (30). Our finding that the expression of c-MYC was important in determining TAK-243 sensitivity is consistent with a recent TAK-243 study in the hematological cancer diffuse large B-cell lymphoma (DLBCL) (44), and conceptually with our recent report with n-MYC (28), where we demonstrated n-MYC sensitizes to NOXA-mediated death in neuroblastoma. We propose here that TAK-243 treatment can capitalize on the MYC effects on ER-stress, by inducing unresolved ER stress preferentially in c-MYC expressing TNBCs, with cells dying through a NOXA-dependent mechanism. While there is an ER-stress response in the sensitive TNBCs without c-MYC expression, it is not as substantial and this correlates with a parallel reduction in cell death and efficacy of TAK-243. Lastly, we found in the normal tissue-derived cells a relatively tempered ER-stress response, which correlates with less toxicity. Thus, c-MYC-dependent ATF4/NOXA induction increases sensitivity of TNBC models to TAK-243 and c-MYC may act as a biomarker for response. Of note, a diagnostic c-MYC immunohistochemical staining has been developed for hematological cancers (45), and this could potentially support the use of c-MYC in future clinical studies of TAK-243 or newer UBA1 inhibitors in TNBC.

Interestingly, and consistent with our findings, other studies support the role of the UPS in TNBC tumor growth: Lieberman and colleagues previously noted the efficacy of proteasome inhibitors in mouse models of TNBC (13). Furthermore, UBR5, the E3 ubiquitin ligase, is overexpressed in TNBC and genetic targeting of UBR5 results in tumor growth inhibition (46). UBR5 is currently not druggable and UBA1 inhibitors may be preferential to proteasome inhibitors for several reasons. First, proteasome inhibitors can be quite toxic to some normal cells (47–49), and while UBA1 inhibitors may also demonstrate normal cell toxicity, our data suggest a therapeutic window. Secondly, Kisselev and colleagues demonstrated targeting additional proteasome sites over what the clinically advanced proteasome inhibitors achieve is necessary for efficacy (50). Thirdly, there is transcriptional feedback to maintain proteasome activity, which can be mediated by nuclear respiratory factor 1 (NRF1) (51). Thus, it appears that TNBCs are vulnerable to the targeting of the UPS and targeting the UPS at the UBA1 stage may offer a better chance of a durable target and a therapeutic window.

In summary, we have demonstrated that targeting UBA1 with TAK-243 in high c-MYC-expressing TNBCs has preclinical activity and may provide an effective therapeutic approach. Thus, TAK-243 may be a valuable targeted therapy approach across diverse TNBC models.

## Material and Methods

### CRISPR screening

TNBC cell lines BT-549 and MDA-MB-468 were infected with a lentiviral genome-wide CRISPR-guide RNA library of unique sgRNAs targeting 20,000 genes (four sgRNAs per gene). Cells were selected in puromycin and cultured for 21 d before isolating genomic DNA and quantifying sgRNA in surviving cells by deep sequencing. This full genomic screen was followed by screening nine TNBCs-cell lines (MDA-MB-468, MDA-MB-231, Hs578T, SUM149PT, BT-549, HCC1806, HCC70, MDA-MB-436, and HCC1937) with 10 sgRNAs against UBA1.

### Cell lines

The TNBC cell lines in this study were cultured in their respective media with 10% FBS in the presence of 1 μg/mL penicillin and streptomycin. BT-549, HCC1937, HCC70, CAL-51, HCC1806, BT20, MDA-MB-157, MDA-MB-436, and RPE1 were grown in RPMI, while MDA-MB-231, MDA-MB-468, HCC38, SUM149PT, and HCE-T were cultured in Dulbecco's Modifed Eagle Medium/Nutrient Mixture F-12 (DMEM/F12). Dulbecco's Modifed Eagle Medium (DMEM) was used to culture CAL-120 and MRC5.

### Reagents and antibodies

TAK-243 (MLN 7243) was purchased from Chemietek (CT-M7243) for in vitro and in vivo studies. The antibodies used in this study were purchased from Cell Signaling and Santa Cruz. All primary antibodies were used at 1:1000 dilutions. Corresponding HRP-conjugated secondary antibodies were used at 1:5000 dilution.

### Cell viability assay

For cell Titer-Glo experiments, 200 to 3000 cells were seeded in 96-well flat-bottom black plates and treated with increasing concentration of drug for  72-h, as previously described ( 55). Following drug treatment, 25 μL of CellTiter-Glo (Promega) was added and read on a Centro LB 960 microplate luminometer (Berthold Technologies). For crystal violet assay, cells were seeded at 50,000 cells/well of 6-well plate. Cells were treated with increasing concentration of drug as indicated and incubated until no treatment wells were confluent. Cells were then fixed with 50% glutaraldehyde and stained with 0.1% crystal violet (Sigma–Aldrich) and visualized. For quantification of crystal violet assay, cells stained with crystal violet were slowly rinsed with PBS prior to addition of 1% SDS to solubilize the crystal violet. The solubilized crystal violet was then quantified at 570 nm using BioTek Gen5 Spectrometer.

### Apoptosis assay

A total of 3 × 10^5^ cells seeded per well in 6-well plates were drugged with the desired concentration of TAK-243 for  24-h or left untreated, as previously described ( 56). Cells were incubated with propidium iodide and annexin V–Cy5 (BD Biosciences) together for 15 min and assayed on a Guava easyCyte flow cytometer (Millipore Sigma). Analysis was performed using FlowJo. Cells stained positive for annexin V-Cy5 and annexin V + propidium were counted as apoptotic.

### Western blot analysis

Cell lysates prepared in NP-40 lysis buffer (20 mM Tris, 150 mM NaCl, 1% Nonidet P-40, 1 mM EDTA, 1 mM EGTA, 10% glycerol, and protease and phosphatase inhibitors) were incubated on ice for 30 min before centrifugation at high speed for 10 min at 4°C. Tumor lysates were homogenized with Tissuemiser (Fisher Scientific) in lysis buffer. Equal amounts of detergent-soluble lysates were resolved using the NuPAGE Novex Midi Gel system on 4% to 12% Bis–Tris gels (Invitrogen). Proteins were transferred to PVDF membranes (PerkinElmer) and blocked in 5% nonfat milk in PBS. Blots were probed with the primary antibodies overnight and later with species appropriate HRP conjugated secondary antibodies. Chemiluminescence was detected with the Syngene G: Box camera (Synoptics).

### Immunohistochemical (IHC) staining

IHC staining for CC3 (1:500, Cell Signaling #9664) was performed in the VCU Tissue and Data Acquisition and Analysis Core with the Leica Bond RX autostainer using heat-induced epitope retrieval buffer 2 (Leica, EDTA pH 8.0) for 20 min with antibody incubation for 45 min. Stained slides were then imaged on the Vectra Polaris (PerkinElmer) and scored. The H-score was determined by multiplication of the percentage of cells with staining intensity ordinal value (3 × % of cells with 3 + intensity) + (2 × % of cells with 2 + intensity) + (1 × % of cells with 1 + intensity) = H-Score.

### Vector construction and stable cell lines

For the shRNA experiments, shATF4, and shATF6 purchased from Dharmacon and the shNOXA previously described (56) were used. shRNA designed against a scramble sequence (MISSION pLKO.1-shRNA control plasmid DNA) served as the control. For the c-MYC over expression, MYC_pLX307 plasmid (Addgene#98,363) and pLX307 empty vector (Addgene#117,734) were used. Cells were transduced with plasmid containing viral particles that were generated in 293T cells and collected over 48 h, as previously described (55). The pLKO.1 puromycin resistant vector backbone served as the basis for cell selection in puromycin following infection. pLenti CRISPR v2 virus against sgRNA targeting c-MYC (Genscript) and pLenti CRISPR v2 virus (control) were commercially made to order from Science Exchange. Cells were then infected with viral particles and selected in puromycin to generate c-MYC KD stable lines.

### PDX and ex vivo studies

All animal experiments were conducted in accordance with a protocol approved by VCU Institutional Animal Care and Use Committee. PDX models HCI-001, WHIM30, and UCD52 were obtained from the University of Utah/Huntsman Cancer Institute, Washington University, St. Louis, and the University of Colorado, respectively, and expanded in NSG mice obtained and bred by VCU Cancer Mouse Models Core (CMMC). The VCU-BC PDX (Supplementary Material Table S4) was developed by VCU CMMC with TNBC tumor samples obtained from VCU Tissue Data Acquisition and Analysis Core (collected under a protocol approved by VCU Institutional Review Board #HM2471). For ex vivo studies, PDX tumors were removed, finely chopped, and digested in DMEM/F12 containing 5% FBS, 300 U/mL collagenase (Sigma) and 100 U/mL hyaluronidase (Sigma). Tissue was then suspended in [(NH_4_)Cl] followed by trypsinization to generate suspensions of single cells, which were previously transduced with lentivirus (BLIV101PA-1, Systems Biosciences) encoding GFP and Luciferase. Cell suspensions were plated at 25,000 cells/well in 96-well plates in M87 medium, followed by treatment with varying concentrations of TAK-243 in triplicate. After  72-h of treatment, luciferin was added to each well (10% of total volume per well) and plates were imaged using the IVIS Spectrum In Vivo Imaging System (PerkinElmer). Cell viability was measured as total photon flux per second, and drug response was evaluated based on % of vehicle viability ( 57). For in vivo studies, 500,000 cells, suspended 1:1 in Matrigel, were injected into the fourth mammary fat pads of experimental female NSG mice. Mice with tumor volumes of ∼150 to 200 mm^3^ were randomized into two groups: TAK-243 treatment and control treatment (vehicle only) and dosed intravenously. TAK-243 was formulated in the vehicle consisting of: 25 mM HCl, 20% 2-hydroxy propyl-β-cyclodextrin (Sigma–Aldrich). Animals were treated with 25 mg/kg of TAK-243 biweekly (Monday and Thursday) for 3 w. Tumors were measured daily by caliper in two dimensions (length and width), and tumor volume was calculated with the formula *v* = *l* × (*w*)2 (π/6), where *v* is the tumor volume, *l* is the length, and *w* is the width (the smaller of the two measurements). Following the final treatment, the tumors were harvested after 2 h and snap frozen in liquid nitrogen for PD studies.

### Experimental metastasis study

MDA-MB-231-tomato-luciferase cells (1 × 10^5^ for endpoint study) in 100-µL sterile PBS were injected into the left cardiac ventricle of female 5-w-old NSG mice as described previously (58) and in vivo imaging was performed (IVIS Spectrum, PerkinElmer) immediately to verify widespread seeding of tumor cells. Mice were randomized using Multi-Task program on day 10 into two groups: TAK-243 treatment and controls and dosed intravenously with TAK-243 formulated in 25 mM HCl, 20% 2-hydroxy propyl-β-cyclodextrin (Sigma–Aldrich) or vehicle alone. Animals were treated with 25 mg/kg of TAK-243 biweekly (Monday and Thursday) for 2 w. Bioluminescence (radiance/sec) emitted from the cells were quantified using the IVIS Spectrum and Living Image software (PerkinElmer) of the animals. For the endpoint study, mice were euthanized, and bioluminescence of harvested organs (kidneys, lungs, ovaries, liver, brain, and skeleton) was quantified by ex vivo imaging and analysis.

## Statistical Analyses

An unpaired student's *t*-test (two-tailed) was used to calculate significance. Differences considered to be significant if *P* < 0.05. For Fig. 3B, linear mixed modeling (lme4 R package v1.1–29) analysis was used to assess treatment effect over time. Mann–Whitney and Welch's unpaired *t*-test was performed for fig. 5A and B and Bonferrroni correction applied.

## Supplementary Material

pgac232_Supplemental_File

## Data Availability

All study data are included in the article and/or Supplementary Material. DepMap data analysis: Gene effect data were obtained from DepMap consortium (https://depmap.org/portal/). The DepMap release contains data from CRISPR knockout screens from project Achilles (52–54), as well as genomic characterization data from the CCLE project. DepMap, Broad (2022): DepMap 22Q2 Public. figshare. Dataset. https://doi.org/10.6084/m9.figshare.19700056.v2
