# Supplementary material for: Genomic screening reveals ubiquitin-like modifier activating enzyme 1 as a potent and druggable target in c-MYC-high triple negative breast cancer models
Source: PNAS Nexus. 2022 Oct 11;1(5):pgac232. doi: 10.1093/pnasnexus/pgac232 (PMC9802478; doi:10.1093/pnasnexus/pgac232)
Supplement: pgac232_Supplemental_File [file pgac232_supplemental_file.pdf]

Table S1. Top 400 genes (approximately 2% of total genes targeted) by STARS scores.

BT549				MDA-MB-468			
Gene Symbol	STARS Score	Average Score	p-value	Gene Symbol	STARS Score	Average Score	p-value
RAD9A	8.25	5.3	1.04E-06	NIP7	8.31	5.14	1.04E-06
RNGTT	8.1	5.3	1.04E-06	C1orf109	8.24	4.98	1.04E-06
SBDS	7.62	5.41	2.08E-06	WDR82	8.23	4.89	1.04E-06
PRC1	7.5	4.81	2.08E-06	MRPS11	8.01	5.28	1.04E-06
BUB3	7.4	4.75	3.12E-06	WDR18	7.75	4.84	2.08E-06
C1orf109	7.29	4.73	4.16E-06	MARS2	7.75	4.89	2.08E-06
PDCD2	7.27	4.57	4.16E-06	TAMM41	7.54	5.13	2.08E-06
LSM2	7.18	4.25	4.16E-06	CRCP	7.51	5.09	2.08E-06
ATP6V0C	7.1	4.71	4.16E-06	NARFL	7.5	5.03	2.08E-06
GINS2	7.08	4.67	4.16E-06	DYNLRB1	7.4	5	3.12E-06
ATP6V1F	7.05	4.42	4.16E-06	FARSB	7.09	4.15	4.16E-06
RFC3	7.02	4.12	4.16E-06	DBR1	6.99	4.99	5.20E-06
TXNL4A	6.99	4.59	5.20E-06	LONP1	6.82	4.47	1.04E-05
WDR82	6.95	4.21	6.24E-06	ZBTB11	6.81	4.05	1.04E-05
HSD17B12	6.92	4.58	7.28E-06	SPATA5L1	6.71	4.79	1.14E-05
CHAF1B	6.91	4.67	7.28E-06	ATP6V1B2	6.68	3.82	1.25E-05
MCM5	6.9	4	7.28E-06	ANKLE2	6.49	4.03	1.56E-05
ACTR1A	6.87	4.57	8.32E-06	SS18L2	6.43	4.64	1.77E-05
TINF2	6.79	3.97	1.04E-05	PMPCB	6.42	3.98	1.98E-05
ELP5	6.77	4.02	1.04E-05	SOD2	6.41	3.84	1.98E-05
PSMD4	6.76	4.17	1.04E-05	ZMAT2	6.3	3.6	2.60E-05
CIRH1A	6.71	4.13	1.14E-05	SCAP	6.29	4.39	2.81E-05
RCC1	6.7	4.38	1.14E-05	YARS2	6.28	4.04	2.91E-05
MRPL33	6.64	4.13	1.35E-05	MMS22L	6.26	3.55	3.12E-05
MAD2L2	6.63	4.01	1.35E-05	SARS	6.25	3.7	3.12E-05
GAPDH	6.62	4.07	1.35E-05	TAF6	6.14	4.46	3.64E-05
YARS	6.6	3.93	1.35E-05	EXOSC8	6.07	3.59	4.26E-05
GTF2H4	6.58	3.94	1.35E-05	PPP1R8	6.03	3.88	4.78E-05
EXOSC6	6.58	4.71	1.46E-05	POLR2L	6.02	3.93	4.89E-05
VPS25	6.5	4.79	1.56E-05	LETM1	6.01	4.41	4.89E-05
NAPG	6.41	3.65	2.08E-05	MAD2L2	5.99	3.62	5.20E-05
SS18L2	6.4	4.45	2.08E-05	CDAN1	5.96	3.71	5.72E-05
MARS2	6.38	4.43	2.18E-05	CYC1	5.93	3.68	5.82E-05
RPL12	6.35	3.82	2.29E-05	BUB3	5.92	4.34	5.82E-05
LONP1	6.35	4.04	2.29E-05	TAF1C	5.91	3.74	5.93E-05
FDXR	6.33	4.09	2.49E-05	HSPE1	5.9	3.36	6.13E-05
DHX37	6.32	3.76	2.60E-05	SNAPC5	5.89	3.71	6.44E-05
NCAPG	6.3	4.22	2.70E-05	RPL23	5.88	3.64	6.44E-05
POLR2L	6.27	3.98	3.01E-05	NOA1	5.87	3.53	6.76E-05
SOD1	6.27	3.98	3.01E-05	CIRH1A	5.85	3.88	6.76E-05
ATP1A1	6.25	3.64	3.12E-05	DPAGT1	5.85	4.37	6.96E-05

YARS2	6.24	5.18	3.12E-05
TOP2A	6.21	3.68	3.43E-05
C9orf114	6.18	3.69	3.64E-05
RFC2	6.17	5.1	3.64E-05
TPI1	6.16	3.53	3.64E-05
POLR3A	6.16	3.97	3.64E-05
DYNLRB1	6.16	4.02	3.64E-05
NOP58	6.15	3.96	3.64E-05
WDR55	6.06	4.01	4.26E-05
TAF1C	6	4.13	5.20E-05
DYNC1I2	6	3.8	5.20E-05
MNAT1	5.98	3.44	5.30E-05
ZMAT2	5.98	3.91	5.41E-05
GPN1	5.97	3.85	5.61E-05
RPL4	5.94	3.94	5.82E-05
DPAGT1	5.93	4.07	5.82E-05
MARS	5.89	3.87	6.34E-05
ESPL1	5.87	4.24	6.44E-05
RPL37	5.87	3.64	6.65E-05
RNMT	5.86	3.7	6.76E-05
NELFB	5.86	4.22	6.76E-05
NHLRC2	5.86	3.4	6.76E-05
MRPS11	5.85	3.71	6.76E-05
GUK1	5.85	4.35	6.76E-05
CPSF4	5.85	3.32	6.96E-05
GGPS1	5.84	3.68	6.96E-05
PRELID1	5.83	4.03	6.96E-05
TARDBP	5.81	3.38	7.07E-05
PWP2	5.81	3.55	7.28E-05
OR4F21	5.8	3.41	7.48E-05
WDR18	5.79	3.83	7.59E-05
LUC7L3	5.77	3.66	7.80E-05
GINS1	5.76	4.26	8.11E-05
GTPBP4	5.75	4.34	8.21E-05
CDC123	5.75	4.3	8.52E-05
RPS3A	5.74	3.62	8.52E-05
TRAPPC3	5.74	3.27	8.52E-05
RPL7	5.73	3.18	8.94E-05
SARS	5.72	3.84	8.94E-05
NCBP2	5.72	4.09	9.15E-05
RGPD8	5.71	3.11	9.25E-05
BIRC5	5.7	3.7	9.67E-05
ARL2	5.69	3.37	9.88E-05
NOL10	5.69	3.76	9.88E-05
HSPE1-MOB4	5.68	3.42	9.88E-05
LRPPRC	5.68	4.06	1.03E-04
EEF2	5.66	3.78	1.06E-04

PELP1	5.77	3.47	7.80E-05
GEMIN5	5.76	3.68	7.80E-05
ALG2	5.74	3.64	8.52E-05
PPP1R2	5.71	4.1	9.15E-05
RFC3	5.71	3.82	9.36E-05
GTF3A	5.71	3.77	9.36E-05
COPS6	5.69	3.96	9.88E-05
RPP40	5.67	3.44	1.03E-04
THOC3	5.64	3.39	1.11E-04
COQ4	5.63	3.25	1.11E-04
PMPCA	5.63	3.61	1.12E-04
NSF	5.62	3.64	1.19E-04
DNAJC9	5.6	3.51	1.26E-04
SOD1	5.6	3.81	1.26E-04
FXN	5.59	3.97	1.27E-04
EARS2	5.55	3.5	1.36E-04
VPS25	5.55	3.97	1.37E-04
SMG6	5.53	3.11	1.46E-04
FBL	5.51	3.71	1.53E-04
TXNL4A	5.5	3.58	1.56E-04
ACTR1A	5.47	3.54	1.65E-04
MRPL18	5.47	3.14	1.66E-04
MVK	5.47	3.46	1.66E-04
RPS4X	5.45	3.48	1.70E-04
TBC1D3C	5.44	3.25	1.77E-04
ATP6V1F	5.42	3.23	1.83E-04
SNAPC1	5.39	3.22	1.91E-04
CPSF4	5.38	3.42	1.98E-04
NDUFAF3	5.37	3.62	2.00E-04
TBC1D3H	5.37	3.23	2.00E-04
SRSF2	5.37	3.27	2.05E-04
NDUFA8	5.34	3.56	2.23E-04
NELFB	5.33	3.91	2.30E-04
COX15	5.33	3.27	2.30E-04
CDC123	5.33	3.32	2.32E-04
RAE1	5.33	2.93	2.34E-04
C1QBP	5.31	3.15	2.48E-04
SF3B5	5.29	3.23	2.59E-04
FNTB	5.29	3.56	2.61E-04
TBC1D3G	5.28	3.04	2.65E-04
TBC1D3	5.28	3.04	2.65E-04
RNPC3	5.27	3.63	2.74E-04
FARS2	5.26	3.51	2.78E-04
INTS3	5.23	3.6	2.95E-04
ROMO1	5.22	3.57	2.98E-04
NDUFB4	5.22	3.1	3.04E-04
CHMP6	5.21	3.76	3.09E-04

EIF2B1	5.65	3.48	1.09E-04
GEMIN5	5.64	3.35	1.10E-04
DDX54	5.63	3.32	1.12E-04
TARS	5.6	3.61	1.23E-04
CCT5	5.58	3.53	1.29E-04
SRP14	5.58	3.99	1.29E-04
CRCP	5.58	3.45	1.30E-04
PMPCA	5.56	3.25	1.34E-04
POLR1C	5.55	4.63	1.35E-04
POLD3	5.55	3.13	1.35E-04
MRPL10	5.52	3.36	1.48E-04
CDC23	5.52	3.51	1.50E-04
DNAJC17	5.5	3.96	1.56E-04
PHB2	5.49	3.58	1.57E-04
MMS22L	5.48	3.5	1.59E-04
NPIPA5	5.48	3.42	1.59E-04
WDHD1	5.47	3.19	1.64E-04
RPL17-C18orf3	5.46	3.24	1.66E-04
CHMP6	5.44	3.77	1.77E-04
MCM6	5.43	3.36	1.80E-04
FBL	5.43	3.61	1.80E-04
SARS2	5.43	3.02	1.82E-04
AFG3L2	5.42	3.41	1.83E-04
MRPL16	5.42	3.26	1.83E-04
DCTN5	5.42	3.25	1.83E-04
RSL1D1	5.38	3.21	1.98E-04
AARS	5.38	3.41	1.99E-04
POLR1A	5.37	3.2	2.00E-04
ALG13	5.37	3.86	2.00E-04
METTL16	5.36	3.16	2.06E-04
SRSF2	5.36	3.36	2.11E-04
CWC22	5.36	3	2.11E-04
DBR1	5.36	4.21	2.11E-04
EARS2	5.35	3.59	2.14E-04
POLD1	5.35	3.04	2.20E-04
HCFC1	5.33	3.77	2.31E-04
SOD2	5.31	3.09	2.48E-04
TRNT1	5.31	4.01	2.48E-04
SAE1	5.3	3.35	2.51E-04
GINS3	5.29	3.83	2.59E-04
GFM1	5.29	3.31	2.61E-04
ALDOA	5.29	3.11	2.62E-04
NSF	5.27	3.21	2.68E-04
COASY	5.26	3.28	2.79E-04
PHF5A	5.26	3.04	2.84E-04
XRCC6	5.25	3.31	2.87E-04
POLR3H	5.25	4.33	2.91E-04

TUBGCP2	5.18	3.47	3.28E-04
PAXBP1	5.17	2.94	3.36E-04
LARS2	5.16	3.14	3.43E-04
POLR3A	5.15	3.12	3.58E-04
CDK7	5.14	3.44	3.63E-04
COG3	5.14	3.17	3.69E-04
GINS2	5.14	3.3	3.72E-04
PRKRA	5.13	3.6	3.77E-04
DDX49	5.09	3.74	4.13E-04
TIMM10	5.08	3.44	4.20E-04
MRPL57	5.07	3.34	4.25E-04
NDNL2	5.06	3.37	4.41E-04
RBM4	5.05	3.11	4.58E-04
HARS2	5.03	2.83	4.89E-04
CCDC115	5.02	3.36	4.95E-04
PET117	5.01	3.81	5.05E-04
MRPL33	4.98	3.51	5.42E-04
RPL9	4.94	3.5	5.95E-04
PDCD2	4.94	3.58	6.01E-04
RPAIN	4.94	3.06	6.01E-04
RBM25	4.92	3.01	6.38E-04
LUC7L3	4.89	3.31	6.83E-04
EEF2	4.89	4.04	6.86E-04
RPS15A	4.89	3.38	6.86E-04
COPG1	4.89	3.37	6.87E-04
ATP5E	4.89	2.98	6.93E-04
SNAPC2	4.89	3.43	6.93E-04
CCT3	4.88	2.77	6.95E-04
PGK1	4.88	3.44	6.95E-04
MYC	4.88	2.95	7.02E-04
MRPL38	4.88	3.7	7.02E-04
UTP15	4.87	2.9	7.05E-04
EEF1A1	4.85	3.15	7.35E-04
PSMG4	4.85	3.35	7.43E-04
HYOU1	4.84	3.38	7.59E-04
VBP1	4.84	3.48	7.61E-04
RIC1	4.84	2.73	7.63E-04
CCDC86	4.83	2.71	7.73E-04
CDC7	4.83	2.79	7.73E-04
TRAPPC8	4.83	3.65	7.77E-04
PSMA3	4.82	2.62	7.88E-04
RPS28	4.82	3.76	7.89E-04
PFDN2	4.82	3.03	8.01E-04
RAD9A	4.77	3.07	9.00E-04
POLR1C	4.76	3.52	9.15E-04
PDCD11	4.76	3.31	9.18E-04
DNAJC17	4.74	3.35	9.55E-04

UBA1	5.24	3.78	2.94E-04
LETM1	5.24	3.82	2.94E-04
SF3A1	5.23	3.52	2.94E-04
RPTOR	5.23	3.27	2.95E-04
NEDD8	5.23	3.03	2.95E-04
EXOSC8	5.22	3.19	3.04E-04
SDHB	5.22	3.38	3.04E-04
UBE2N	5.22	3.65	3.04E-04
KIF18A	5.21	3.51	3.04E-04
RABGGTB	5.2	3.4	3.11E-04
MRPL28	5.2	3.1	3.11E-04
CIAO1	5.2	3.64	3.20E-04
TONSL	5.19	3.71	3.21E-04
MLST8	5.19	3.05	3.27E-04
PSMB6	5.19	2.96	3.27E-04
MVK	5.18	3.27	3.28E-04
IPO9	5.18	3.19	3.30E-04
CDC45	5.18	2.93	3.33E-04
DDX55	5.17	3.7	3.35E-04
SNRNP25	5.17	3.76	3.35E-04
PSMG4	5.17	3.52	3.35E-04
FCF1	5.17	3.36	3.41E-04
TTI1	5.16	2.89	3.42E-04
RUVBL1	5.16	3.76	3.43E-04
EIF1AX	5.15	3.7	3.62E-04
ORAOV1	5.14	3.72	3.67E-04
UBE2L3	5.14	3.19	3.69E-04
CCT3	5.13	3.12	3.77E-04
URB1	5.13	3.17	3.77E-04
RBM22	5.13	2.78	3.84E-04
MAK16	5.12	3.4	3.89E-04
POLRMT	5.12	3.07	3.89E-04
NOP2	5.12	3.07	3.94E-04
HDAC3	5.11	3.2	3.96E-04
RPAP1	5.11	3.73	3.96E-04
OSGEP	5.11	3.45	3.97E-04
TRMT112	5.1	3.7	4.02E-04
ANKLE2	5.1	3.48	4.02E-04
CCDC84	5.09	3.2	4.15E-04
EIF3I	5.08	2.85	4.20E-04
TEN1	5.06	3.19	4.41E-04
PGK1	5.06	3.11	4.51E-04
RPL31	5.04	3.12	4.66E-04
LYRM4	5.04	3.37	4.78E-04
LARS2	5.03	3.16	4.88E-04
SNAPC5	5.03	3.2	4.92E-04
RPL34	5.02	2.99	5.02E-04

RPL19	4.73	2.92	9.78E-04
RPL35	4.72	2.92	9.86E-04
CENPW	4.71	2.94	1.01E-03
FAM96B	4.71	3.25	1.02E-03
DHX33	4.71	3.39	1.02E-03
HEATR1	4.7	2.99	1.03E-03
MTPAP	4.7	3.08	1.03E-03
HNRNPM	4.69	3.66	1.06E-03
XRCC3	4.69	3.3	1.07E-03
YARS	4.68	2.8	1.07E-03
RNGTT	4.68	3.26	1.07E-03
POP5	4.68	3.53	1.08E-03
DHX37	4.67	3.13	1.11E-03
SAE1	4.67	3.4	1.11E-03
NDUFB11	4.66	2.94	1.14E-03
CAP1	4.65	3.3	1.16E-03
MIPEP	4.65	3.01	1.16E-03
NDUFB10	4.65	3.23	1.16E-03
NDUFAF1	4.64	3.04	1.19E-03
CHAF1B	4.64	3.2	1.20E-03
SBDS	4.63	3.56	1.21E-03
NOP9	4.63	3.6	1.22E-03
SMU1	4.62	2.94	1.24E-03
CYCS	4.61	3.11	1.29E-03
UQCRC2	4.61	3.05	1.29E-03
MCM4	4.61	2.96	1.29E-03
SRBD1	4.6	2.74	1.30E-03
RUVBL2	4.6	2.71	1.31E-03
HSPE1-MOB4	4.6	2.98	1.31E-03
CTCF	4.6	3.66	1.32E-03
EMC3	4.56	2.75	1.43E-03
POLR3H	4.56	3.71	1.44E-03
MRPS23	4.55	3.03	1.46E-03
TKT	4.55	2.77	1.47E-03
DCTN5	4.54	2.8	1.48E-03
TUBB	4.54	2.91	1.48E-03
C21orf59	4.52	3.2	1.57E-03
BOP1	4.52	3.12	1.59E-03
POLD3	4.51	2.65	1.60E-03
C3orf17	4.51	3.16	1.60E-03
H2AFX	4.51	3.16	1.61E-03
PSMB6	4.51	2.57	1.62E-03
NDUFB6	4.5	3.8	1.66E-03
LRR1	4.49	3.08	1.67E-03
GNL3	4.49	3.11	1.69E-03
GINS1	4.49	3.33	1.69E-03
BRIX1	4.48	2.8	1.72E-03

PKM	5.02	3.23	5.05E-04
MCM3	5.01	3.78	5.05E-04
CYCS	5.01	2.9	5.14E-04
DDX10	5.01	3.39	5.15E-04
ATP6V1B2	5.01	3.8	5.18E-04
SFSWAP	5	3.33	5.24E-04
PSMA5	4.99	2.73	5.26E-04
PRMT1	4.99	3.68	5.28E-04
SF3A2	4.97	2.84	5.47E-04
RABGGTA	4.97	3.54	5.48E-04
BUB1B	4.97	2.93	5.49E-04
PPA1	4.96	3.29	5.76E-04
PSMA1	4.95	3.03	5.80E-04
MYC	4.95	2.9	5.83E-04
MRPL21	4.95	2.96	5.93E-04
RPL23	4.95	3.15	5.94E-04
C3orf17	4.94	2.85	5.99E-04
NPLOC4	4.94	3.27	6.07E-04
GTF2A2	4.92	3.36	6.26E-04
INTS3	4.92	3.72	6.30E-04
SNRPG	4.91	3.38	6.39E-04
CDCA8	4.91	3.16	6.39E-04
FARSB	4.9	4.43	6.66E-04
MRPS23	4.9	3.52	6.66E-04
SPATA5L1	4.9	3.19	6.74E-04
SF1	4.9	2.9	6.76E-04
MRPL39	4.89	3.53	6.87E-04
THOC3	4.89	3.31	6.93E-04
RBBP5	4.88	3.27	6.95E-04
DDB1	4.88	3.05	6.95E-04
RPL14	4.88	3.17	7.03E-04
RPL24	4.88	2.89	7.04E-04
E2F3	4.88	2.91	7.04E-04
PIK3C3	4.87	2.85	7.06E-04
DHPS	4.87	3.06	7.16E-04
CCT2	4.86	3.24	7.22E-04
METTL14	4.85	2.91	7.34E-04
NVL	4.85	3.28	7.43E-04
CCT4	4.85	2.95	7.44E-04
TOP3A	4.84	2.87	7.45E-04
RPL27A	4.84	3.27	7.60E-04
NMD3	4.84	3.05	7.61E-04
VCP	4.83	2.85	7.71E-04
IARS2	4.83	4.04	7.73E-04
CENPN	4.83	2.75	7.73E-04
OBFC1	4.83	3.33	7.73E-04
DDX47	4.83	3.04	7.75E-04

DTYMK	4.48	3.37	1.72E-03
LRPPRC	4.48	2.68	1.73E-03
DERL2	4.47	2.73	1.74E-03
INTS7	4.47	3.09	1.77E-03
SDHB	4.47	3.41	1.77E-03
TARDBP	4.47	2.62	1.78E-03
BUD31	4.46	2.79	1.82E-03
NUP88	4.45	2.87	1.85E-03
PPCDC	4.45	2.79	1.86E-03
RPL11	4.44	3.19	1.87E-03
DDX56	4.44	2.67	1.88E-03
RBM33	4.44	2.73	1.91E-03
EIF2B3	4.44	2.86	1.91E-03
UMPS	4.43	2.91	1.92E-03
NHLRC2	4.43	3.03	1.93E-03
NCAPG2	4.43	2.39	1.95E-03
RBBP5	4.42	2.88	1.97E-03
TARS2	4.42	3.07	1.99E-03
NDUFAB1	4.41	2.84	2.03E-03
ORAOV1	4.41	2.82	2.03E-03
ATP6V0C	4.4	3.38	2.06E-03
AARS	4.4	3.21	2.06E-03
FAU	4.4	2.63	2.07E-03
EPRS	4.38	2.66	2.14E-03
RUVBL1	4.38	3.49	2.16E-03
NAE1	4.38	2.81	2.17E-03
COA7	4.36	2.72	2.26E-03
MRPS31	4.36	3.06	2.26E-03
EIF1AX	4.36	2.8	2.28E-03
GLRX5	4.35	2.97	2.29E-03
PDRG1	4.35	2.92	2.30E-03
YRDC	4.35	2.86	2.30E-03
FTSJ2	4.35	2.9	2.32E-03
UBE2N	4.32	2.88	2.47E-03
MAK16	4.32	2.65	2.49E-03
UBA1	4.32	2.72	2.50E-03
MRPL43	4.32	2.78	2.51E-03
SPATA5	4.3	2.99	2.60E-03
KANSL3	4.3	2.54	2.61E-03
MINOS1	4.3	2.99	2.64E-03
MED30	4.29	2.98	2.65E-03
POLR3E	4.29	3.26	2.66E-03
RPL4	4.29	2.79	2.70E-03
MRPL15	4.28	2.81	2.71E-03
PAFAH1B1	4.28	2.89	2.75E-03
KIAA0391	4.27	2.74	2.76E-03
MED19	4.27	2.57	2.77E-03

MRGBP	4.82	3.19	7.91E-04
CDC42	4.82	3.43	8.00E-04
UBA2	4.82	2.67	8.01E-04
PPP4C	4.81	3.2	8.05E-04
C10orf2	4.81	2.89	8.10E-04
MRPL20	4.81	2.97	8.14E-04
SMC3	4.81	3.15	8.14E-04
PSMD14	4.8	2.71	8.22E-04
RPS13	4.8	3.58	8.31E-04
C21orf59	4.8	3.3	8.31E-04
SART3	4.8	2.87	8.38E-04
CPSF3L	4.79	3.31	8.42E-04
ERH	4.78	3.48	8.68E-04
HSPD1	4.78	3.27	8.84E-04
GNB2L1	4.77	3.13	8.88E-04
SYS1	4.76	3.1	9.13E-04
RPLP0	4.76	3.24	9.15E-04
CTCF	4.76	3.45	9.15E-04
RACGAP1	4.76	3.28	9.18E-04
EIF2B3	4.76	2.94	9.20E-04
MPHOSPH10	4.75	2.88	9.23E-04
UBE2I	4.75	3.43	9.23E-04
TOMM22	4.75	2.86	9.23E-04
SDHC	4.75	2.96	9.40E-04
SUPV3L1	4.74	3.13	9.55E-04
MMS19	4.74	3.07	9.66E-04
SUPT16H	4.73	2.77	9.68E-04
C1QBP	4.73	2.72	9.77E-04
RPS8	4.73	2.77	9.78E-04
MRPS6	4.73	2.74	9.78E-04
DDX11	4.73	2.76	9.84E-04
TBCB	4.72	2.92	9.91E-04
PSMA6	4.72	2.74	1.00E-03
RPL18	4.71	3.03	1.01E-03
RPL7A	4.71	2.67	1.01E-03
CDIPT	4.71	3.39	1.02E-03
PGD	4.71	3.06	1.02E-03
PAM16	4.71	3.14	1.03E-03
PDRG1	4.7	3.32	1.03E-03
KRR1	4.7	3.53	1.03E-03
RPL9	4.7	3.47	1.03E-03
PRPF19	4.69	3.34	1.06E-03
MRPL49	4.68	2.75	1.07E-03
UTP15	4.68	2.75	1.09E-03
PDCD11	4.67	3.34	1.10E-03
EEF1G	4.67	3.52	1.10E-03
TRAPPC1	4.66	2.92	1.14E-03

PFDN1	4.27	2.9	2.79E-03
CLNS1A	4.27	2.8	2.79E-03
MASTL	4.27	2.96	2.81E-03
PRPF38B	4.26	2.99	2.83E-03
TARS	4.26	2.89	2.86E-03
ARMC7	4.26	2.99	2.86E-03
TRMT112	4.26	3.16	2.87E-03
TIMELESS	4.26	2.58	2.87E-03
RPUSD3	4.25	2.93	2.91E-03
TCP1	4.25	2.83	2.95E-03
MRPS34	4.24	3.26	2.98E-03
SNRPD1	4.24	2.56	2.98E-03
DDX55	4.24	2.45	3.01E-03
RNF4	4.23	2.31	3.05E-03
GPN2	4.23	3.19	3.06E-03
CCT4	4.22	3.01	3.12E-03
MTG2	4.21	2.78	3.18E-03
MRPL10	4.21	3.01	3.19E-03
NKAP	4.21	2.74	3.21E-03
USP9X	4.19	2.58	3.31E-03
TOP2A	4.19	2.38	3.32E-03
RABIF	4.18	2.29	3.39E-03
COASY	4.18	3.23	3.39E-03
PCNA	4.18	3.38	3.39E-03
DDX10	4.18	2.77	3.41E-03
PRPF3	4.18	2.48	3.42E-03
CAPZB	4.18	2.75	3.43E-03
NUP43	4.18	2.86	3.44E-03
CCNH	4.18	3.06	3.44E-03
CDK1	4.17	2.6	3.51E-03
GARS	4.17	2.57	3.52E-03
TUBG1	4.17	2.7	3.53E-03
ATL2	4.16	2.36	3.59E-03
DHDDS	4.16	2.59	3.61E-03
TBC1D3F	4.15	2.46	3.70E-03
CDC37	4.14	3.03	3.74E-03
RPL27	4.14	2.55	3.76E-03
RPS8	4.14	2.99	3.79E-03
RPL36	4.13	3.02	3.79E-03
C9orf114	4.13	2.76	3.82E-03
EXOSC1	4.13	2.6	3.87E-03
RABGGTA	4.13	2.73	3.89E-03
PSTK	4.12	2.84	3.91E-03
RPAP1	4.12	2.92	3.91E-03
NDUFA2	4.12	2.36	3.92E-03
ERAL1	4.11	2.64	4.03E-03
MED11	4.11	2.6	4.05E-03

CSE1L	4.65	2.9	1.17E-03
PPWD1	4.65	3.25	1.18E-03
RFC5	4.64	3.36	1.19E-03
GRB2	4.64	2.63	1.20E-03
HAUS7	4.64	3	1.20E-03
MRPS25	4.63	2.99	1.21E-03
EEF1A1	4.63	2.77	1.22E-03
BNIP1	4.63	2.78	1.23E-03
SNRPF	4.63	3.35	1.23E-03
ELL	4.63	2.99	1.23E-03
NUP133	4.61	2.69	1.29E-03
XPO1	4.6	3.19	1.30E-03
NFS1	4.6	3.49	1.30E-03
PKMYT1	4.6	2.78	1.32E-03
WDR43	4.6	2.97	1.32E-03
SKP2	4.59	2.59	1.34E-03
EIF2S1	4.59	2.92	1.34E-03
ETF1	4.59	2.76	1.35E-03
SNF8	4.59	2.87	1.35E-03
WDR74	4.58	2.88	1.37E-03
ACTL6A	4.57	3.02	1.39E-03
RPS20	4.57	3.04	1.41E-03
RGPD6	4.57	2.58	1.41E-03
RGPD5	4.57	2.58	1.41E-03
SPC24	4.57	3.71	1.42E-03
ECT2	4.56	3.02	1.42E-03
PNPT1	4.56	3.03	1.42E-03
UBA3	4.56	2.83	1.43E-03
GOSR2	4.56	2.48	1.44E-03
TXN	4.56	3.42	1.44E-03
SNUPN	4.56	2.82	1.44E-03
DENR	4.55	2.74	1.45E-03
ING3	4.55	2.75	1.46E-03
WRB	4.55	2.65	1.46E-03
RPA1	4.54	2.92	1.48E-03
DYNLL1	4.54	2.89	1.49E-03
TSG101	4.54	2.65	1.50E-03
PSMA3	4.53	2.89	1.52E-03
MRPL53	4.53	2.94	1.52E-03
CFAP20	4.53	2.53	1.53E-03
SF3A3	4.53	2.85	1.53E-03
PSMA7	4.52	2.93	1.55E-03
ITGAV	4.52	3.12	1.57E-03
NUDT21	4.52	2.95	1.57E-03
RRM1	4.52	3	1.57E-03
AARS2	4.51	2.76	1.61E-03
RPS29	4.51	2.69	1.61E-03

RPN2	4.11	3.26	4.05E-03
WDR48	4.1	2.47	4.13E-03
C12orf65	4.1	2.62	4.18E-03
DNM1L	4.08	2.92	4.40E-03
DDX42	4.07	2.59	4.44E-03
GGPS1	4.07	2.37	4.45E-03
SRP14	4.07	2.74	4.51E-03
MYBBP1A	4.06	3.09	4.55E-03
SKP2	4.06	2.36	4.55E-03
KIF18A	4.06	2.78	4.57E-03
UTP3	4.06	2.67	4.58E-03
IARS2	4.05	3.18	4.64E-03
ENO1	4.05	2.68	4.71E-03
DONSON	4.04	2.6	4.80E-03
HAUS7	4.03	3.07	4.94E-03
CIAO1	4.03	2.89	4.95E-03
POLG2	4.02	2.44	5.05E-03
TANGO6	4.01	3.06	5.06E-03
C10orf2	4.01	2.52	5.14E-03
MRPL21	4	2.88	5.20E-03
GNB2L1	4	3.32	5.25E-03
MRPL2	4	2.31	5.27E-03
ATP6V1G1	3.99	2.55	5.34E-03
POLR3D	3.98	2.65	5.40E-03
USP5	3.98	2.86	5.41E-03
RAD51D	3.98	2.66	5.42E-03
XPO1	3.97	2.77	5.43E-03
RBBP4	3.97	2.67	5.46E-03
PGS1	3.97	2.57	5.46E-03
MRPS24	3.96	3.27	5.51E-03
DHPS	3.96	3	5.54E-03
ADSL	3.95	2.83	5.68E-03
MRPL4	3.93	2.56	5.79E-03
DDB1	3.93	3.1	5.79E-03
SNRNP35	3.89	2.61	6.21E-03
SEPHS2	3.88	3.12	6.26E-03
ELP5	3.87	2.75	6.43E-03
SMC1A	3.86	2.8	6.46E-03
HIST2H3A	3.85	2.37	6.64E-03
HIST2H3C	3.85	2.37	6.64E-03
MVD	3.84	2.49	6.69E-03
PRPF19	3.84	2.82	6.70E-03
EEF1G	3.84	2.87	6.76E-03
MRTO4	3.83	2.82	6.80E-03
HAUS5	3.83	2.98	6.82E-03
ANKRD49	3.83	2.36	6.84E-03
ZNF131	3.83	2.88	6.86E-03

CHMP4B	4.51	3.26	1.62E-03
RPL19	4.51	2.82	1.62E-03
NUP85	4.51	2.91	1.62E-03
FAU	4.5	3.03	1.63E-03
EEF2KMT	4.5	3.14	1.63E-03
POP5	4.5	2.99	1.64E-03
NAE1	4.49	2.95	1.67E-03
NAA10	4.49	3.22	1.69E-03
VRK1	4.48	2.9	1.70E-03
DDX59	4.48	2.72	1.70E-03
SNAPC1	4.48	2.78	1.72E-03
SNRPE	4.48	3.29	1.73E-03
ALG2	4.48	3.01	1.73E-03
ATP6V1A	4.48	2.85	1.74E-03
RPL3	4.47	2.85	1.74E-03
NOL9	4.47	3.08	1.76E-03
TSR2	4.47	3.09	1.76E-03
RPS6	4.47	2.78	1.76E-03
MASTL	4.47	3.25	1.78E-03
RAN	4.46	2.62	1.78E-03
NARS	4.46	2.64	1.80E-03
PSMA2	4.46	2.79	1.81E-03
DHX33	4.46	3	1.81E-03
DCLRE1B	4.46	2.72	1.82E-03
RPL5	4.45	3.18	1.82E-03
RPS19	4.45	3.29	1.82E-03
FAM96B	4.45	2.85	1.84E-03
ICE2	4.45	2.6	1.86E-03
GTF2E1	4.44	3.06	1.89E-03
TIMM13	4.44	2.44	1.89E-03
LSM3	4.44	3.23	1.90E-03
SRSF7	4.44	3.37	1.91E-03
SEC13	4.43	2.62	1.93E-03
MRPL22	4.43	2.7	1.93E-03
SPCS3	4.43	3.28	1.94E-03
DDX49	4.43	2.85	1.95E-03
SRBD1	4.41	3.39	2.00E-03
SRSF1	4.41	2.54	2.02E-03
KIAA0391	4.4	2.95	2.05E-03
POLE	4.4	3.03	2.05E-03
TIMM10	4.4	3.03	2.07E-03
CDK7	4.39	3.6	2.08E-03
TUBGCP5	4.39	2.54	2.08E-03
WDR61	4.39	2.88	2.10E-03
IMP4	4.39	2.74	2.10E-03
NPIPA2	4.39	3.17	2.12E-03
NPIPA1	4.39	3.17	2.12E-03

SHQ1	3.82	2.43	6.93E-03
TBCB	3.81	2.89	7.04E-03
NUDC	3.8	2.48	7.24E-03
RPL12	3.79	2.77	7.27E-03
NMT1	3.79	3.12	7.32E-03
GNB2	3.79	2.44	7.36E-03
GTPBP4	3.78	3.13	7.40E-03
DDOST	3.78	2.77	7.40E-03
RARS2	3.78	2.78	7.44E-03
RPP21	3.78	2.57	7.44E-03
PLA2G10	3.76	2.51	7.74E-03
NDUFA1	3.75	2.47	7.87E-03
AK6	3.75	2.88	7.88E-03
EP400	3.75	2.4	7.92E-03
MRPL23	3.74	2.65	7.93E-03
SF3A2	3.74	2.31	8.06E-03
ACTR2	3.74	2.85	8.06E-03
TELO2	3.73	2.31	8.21E-03
SLC52A1	3.73	2.58	8.23E-03
GTF2H1	3.71	2.69	8.40E-03
MCM6	3.71	2.43	8.44E-03
KPNB1	3.71	2.47	8.46E-03
RRS1	3.7	2.84	8.57E-03
IGBP1	3.69	2.64	8.85E-03
TIMM13	3.69	2.44	8.86E-03
UQCRFS1	3.68	2.33	8.94E-03
NOP58	3.68	2.32	8.97E-03
PAM16	3.68	2.51	9.01E-03
MIS18A	3.67	2.83	9.04E-03
CUL1	3.67	2.61	9.04E-03
TRIT1	3.67	2.68	9.16E-03
MED6	3.66	2.47	9.28E-03
PRMT5	3.65	2.78	9.44E-03
URB1	3.64	2.79	9.66E-03
IMP3	3.64	2.86	9.70E-03
MRPL16	3.64	2.92	9.76E-03
ATP6V1A	3.63	2.82	9.81E-03
BCLAF1	3.63	2.3	9.92E-03
TRIAP1	3.62	2.65	9.98E-03
HSD17B10	3.62	2.34	1.01E-02
UQCC2	3.6	2.65	1.05E-02
LANCL2	3.59	2.27	1.06E-02
CSNK2B	3.59	2.32	1.07E-02
RMI1	3.58	2.57	1.08E-02
NOL10	3.58	2.76	1.09E-02
COX17	3.57	2.25	1.10E-02
ALYREF	3.57	2.26	1.12E-02

NPIPA3	4.39	3.17	2.12E-03
KANSL3	4.38	2.39	2.17E-03
NOP10	4.37	2.5	2.19E-03
CDC37	4.37	2.98	2.20E-03
RPL17	4.37	2.79	2.21E-03
HUS1	4.37	2.8	2.21E-03
RPL35A	4.36	3.05	2.23E-03
SMG1	4.36	2.73	2.24E-03
MDN1	4.36	3.2	2.24E-03
PGAM1	4.36	2.51	2.25E-03
GTF2B	4.36	3.02	2.28E-03
XRN2	4.36	2.82	2.28E-03
DAD1	4.35	3.57	2.30E-03
ACTR10	4.35	2.59	2.30E-03
PRMT5	4.35	3.26	2.30E-03
RPL6	4.35	3.62	2.30E-03
KPNB1	4.35	2.75	2.32E-03
TRRAP	4.35	2.62	2.34E-03
ATP2A2	4.34	3.41	2.36E-03
OXSM	4.34	2.46	2.37E-03
RRP12	4.34	2.78	2.38E-03
RPS7	4.33	2.55	2.39E-03
MED11	4.33	3.15	2.40E-03
GNB1L	4.33	2.64	2.40E-03
FEN1	4.33	2.35	2.43E-03
EIF1AD	4.32	3.14	2.46E-03
CARS	4.32	3.11	2.46E-03
POLE2	4.32	2.7	2.50E-03
MCM7	4.32	2.85	2.51E-03
RPS16	4.31	2.62	2.55E-03

PSMD7	3.56	2.64	1.12E-02
TBCA	3.56	3.36	1.14E-02
CPSF6	3.55	2.48	1.15E-02
ATP5I	3.55	2.68	1.15E-02
HJURP	3.55	2.25	1.15E-02
ZRSR2	3.55	2.58	1.16E-02
PPIL4	3.55	2.49	1.16E-02
EIF2S1	3.54	2.58	1.17E-02
HAUS1	3.54	2.84	1.18E-02
NFRKB	3.54	2.41	1.19E-02
BIRC6	3.54	2.58	1.19E-02
PPP1R12A	3.53	2.36	1.19E-02
DCTN3	3.53	2.55	1.20E-02
EEF2KMT	3.52	2.68	1.23E-02
NAA10	3.52	2.91	1.24E-02
OXSM	3.51	2.19	1.25E-02
RPS19	3.51	2.82	1.26E-02
NOL6	3.5	2.56	1.28E-02
SEPSECS	3.49	2.83	1.30E-02
CCDC51	3.48	2.46	1.32E-02
RCC1	3.48	2.7	1.34E-02
TTF2	3.48	2.34	1.35E-02
SETD1A	3.47	2.44	1.36E-02
SNRNP27	3.47	2.22	1.36E-02
MCM3	3.47	2.77	1.37E-02
TXN	3.47	2.34	1.37E-02
RPS20	3.46	2.62	1.39E-02
MCMBP	3.46	2.21	1.40E-02
DAD1	3.45	2.9	1.41E-02
WDR43	3.45	2.81	1.43E-02

Table S2. Ubiquitination related hits and their scores in the primary screen analysis.

BT-549

Gene Symbol	STARS Score	Average Score	p-value
PSMD4	6.8	4.2	1.04E-05
UBA1	5.2	3.8	2.95E-04
NEDD8	5.2	3.0	2.95E-04
PSMB6	5.2	3.0	3.27E-04
PSMA5	5.0	2.7	5.26E-04
PSMA1	5.0	3.0	5.80E-04
UBA2	4.8	2.7	8.01E-04
PSMD14	4.8	2.7	8.22E-04
PSMA6	4.7	2.7	1.00E-03
UBA3	4.6	2.8	1.43E-03
PSMA3	4.5	2.9	1.51E-03
PSMA7	4.5	2.9	1.54E-03
PSMA2	4.5	2.8	1.80E-03

MDA-MB-468

Gene Symbol	STARS Score	Average Score	p-value
PSMA3	4.82	2.6	7.88E-04
PSMB6	4.51	2.6	1.61E-03
UBA1	4.32	2.7	2.50E-03
RNF4	4.23	2.3	3.05E-03
USP9X	4.19	2.6	3.31E-03
USP5	3.98	2.9	5.41E-03
PSMD7	3.56	2.6	1.12E-02

Table S3. UBA1 and ubiquitination related genes and their CERES scores in TNBC CRISPR knock out cell lines derived from DepMap portal.

Genes	Cell lines													
	Hs578T	CAL120	HCC1937	DU4475	HCC38	BT549	MDA-MB-436	MDA-MB-157	HCC1806	HCC70	MDA-MB-231	MDA-MB-468	CAL51	SUM149PT
UBA1	-2.27	-2.01	-2.26	-2.21	-2.28	-2.33	-2.02	-1.73	-2.07	-2.25	-2.04	-2.08	-2.26	-1.68
UBA2	-0.83	-0.65	-1.06	-1.00	-0.73	-0.87	-1.01	-1.11	-1.35	-0.90	-1.26	-1.42	-1.17	-1.00
UBA3	-0.71	-0.82	-0.71	-1.32	-0.80	-1.10	-0.56	-0.89	-1.10	-0.62	-0.69	-0.77	-1.02	-0.77
NEDD8	-1.52	-2.16	-2.00	-1.00	-1.72	-1.74	-1.63	-2.21	-1.92	-1.90	-1.74	-1.75	-1.47	-1.80
PSMD4	-1.74	-1.67	-1.46	-1.33	-2.07	-1.60	-1.14	-1.84	-1.76	-1.32	-1.41	-1.55	-1.28	-0.84
PSMB6	-1.95	-1.58	-1.52	-2.33	-1.55	-1.44	-1.76	-0.49	-1.16	-0.30	-1.25	-1.12	-1.74	-0.97
PSMA1	-2.23	-2.41	-2.28	-1.98	-2.23	-2.41	-2.44	-2.62	-2.18	-2.10	-2.27	-2.19	-2.15	-2.19
PSMD14	-1.75	-1.70	-2.05	-2.14	-1.92	-1.75	-1.93	-2.13	-1.82	-2.25	-1.71	-1.86	-1.39	-1.07
PSMA6	-2.33	-2.49	-2.53	-2.41	-2.34	-2.37	-2.39	-2.53	-2.35	-2.31	-2.40	-2.35	-2.36	-1.97
PSMA3	-2.32	-2.47	-2.46	-2.46	-1.99	-2.33	-2.36	-2.56	-2.09	-2.29	-2.33	-2.22	-2.20	-2.31
PSMA7	-2.23	-2.12	-2.16	-2.13	-2.12	-1.88	-2.08	-1.77	-1.91	-2.29	-2.15	-2.11	-1.99	-1.96
PSMD7	-1.96	-1.75	-1.91	-1.66	-1.85	-1.83	-1.96	-1.80	-1.79	-1.55	-1.62	-1.64	-1.32	-1.63
RNF4	-0.63	-0.18	-0.53	-1.06	-0.99	-0.16	-0.49	-0.98	-0.95	-0.44	-0.35	-0.78	-0.95	-0.48
USP9X	-0.04	-0.39	-0.68	-0.88	-0.73	-0.38	-0.68	-0.27	-0.63	-0.20	-0.12	-0.36	-0.05	-0.77
USP5	-1.64	-1.94	-1.66	-1.80	-2.09	-1.95	-1.60	-1.91	-1.97	-1.33	-1.64	-1.89	-1.80	-2.15

Table S4: Details of VCU PDX models.

Description	Age	Diagnosis
VCU-BC-01	59	Invasive Ductal Carcinoma Grade III ER-, PR-, Her2Neu- (Triple negative)
VCU-BC-02	52	Invasive ductal carcinoma, ER-, PR-, HER2Neu- (Triple Negative)
VCU-BC-03	53	Invasive ductal carcinoma with necrosis, ER-, PR-, HER2Neu-

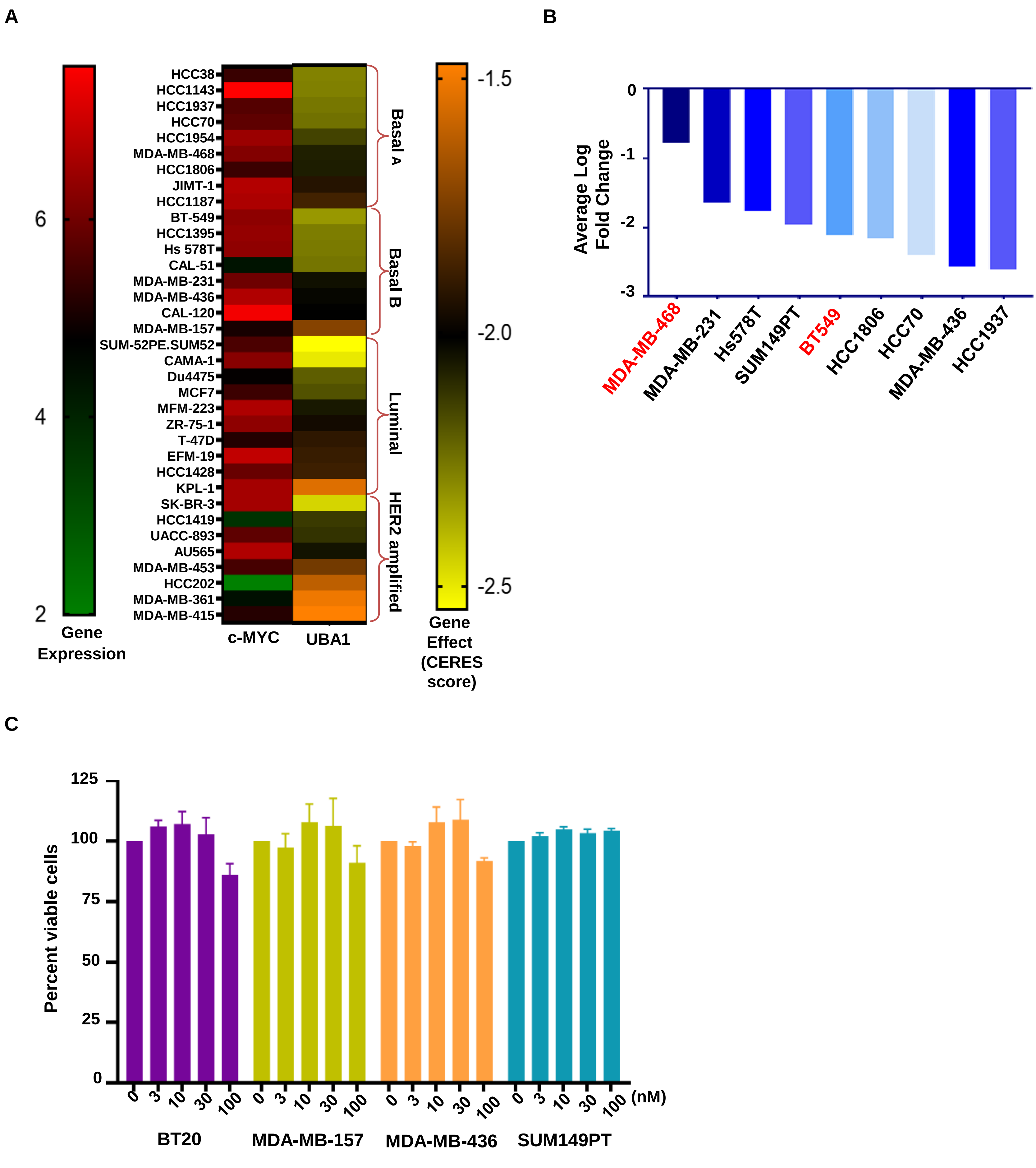

Figure S1. UBA1 expression levels in TNBC lines. (A) Heat map represents CERES score of UBA1 gene in CRISPR knock out cells and c-MYC expression obtained in various breast cancer models. (B) Graph represents average Log Fold Change (LFC) in the expression of guide RNAs against UBA1 in nine TNBC cell lines. Average LFC was calculated from LFC of 10 clones/sgRNA of UBA1 in the CRISPR analysis. (C) Graph represents percent viable cells assessed by CellTiter-Glo in TNBCs lines BT-20, MDA-MB-157, MDA-MB-436 and SUM149PT following 72 h treatment with TAK-243 at the indicated concentration.

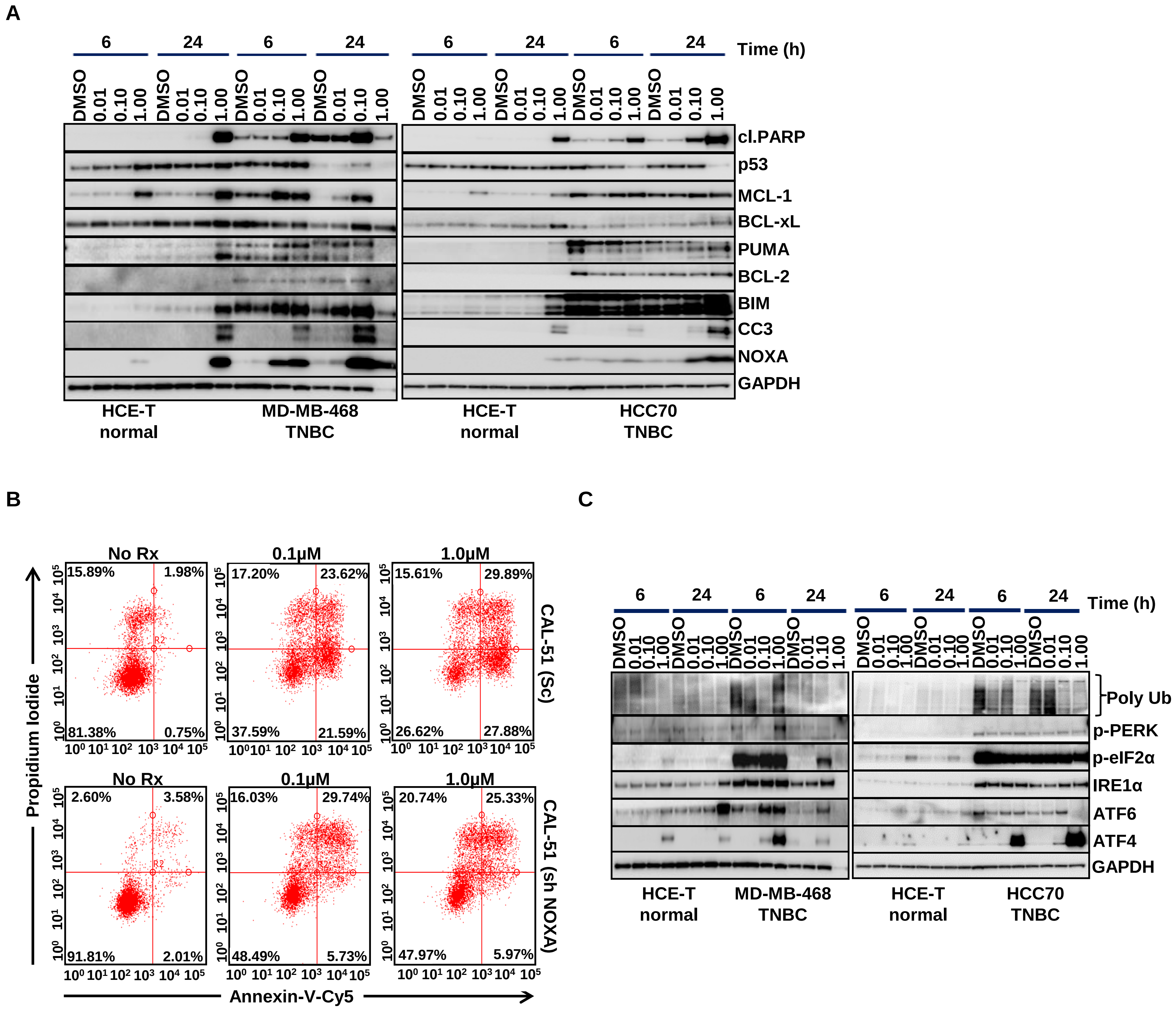

Fig S2. TAK-243 induced cell death in TNBC. (A) Western blot analysis showing dose response and time course of the effects of TAK-243 on ubiquitination and apoptosis in TNBC (MDA-MB-468 and HCC70) and normal tissue-derived HCE-T cells, as assessed by immunoblotting for p53, MCL-1, Bcl-xL, BIM, PUMA, cleaved Caspase 3 (CC3) and NOXA. GAPDH was used as a loading control. (B) FACS analysis demonstrating Annexin-V-Cy5 and Propidium Iodide staining in pLKO.1-shRNA control and CAL-51 NOXA knockdown stable cells following 24h treatment with TAK-243 at the indicated concentrations. (C) Western blot analysis showing dose response and time course of the effects of TAK-243 on UPR proteins in TNBC (MDA-MB-468 and HCC70) and normal tissue-derived HCE-T cells, as assessed by immunoblotting for ATF4 and ATF6. Polyubiquitin (polyUb) indicates ubiquitin engagement in these cells. GAPDH was used as a loading control.

A

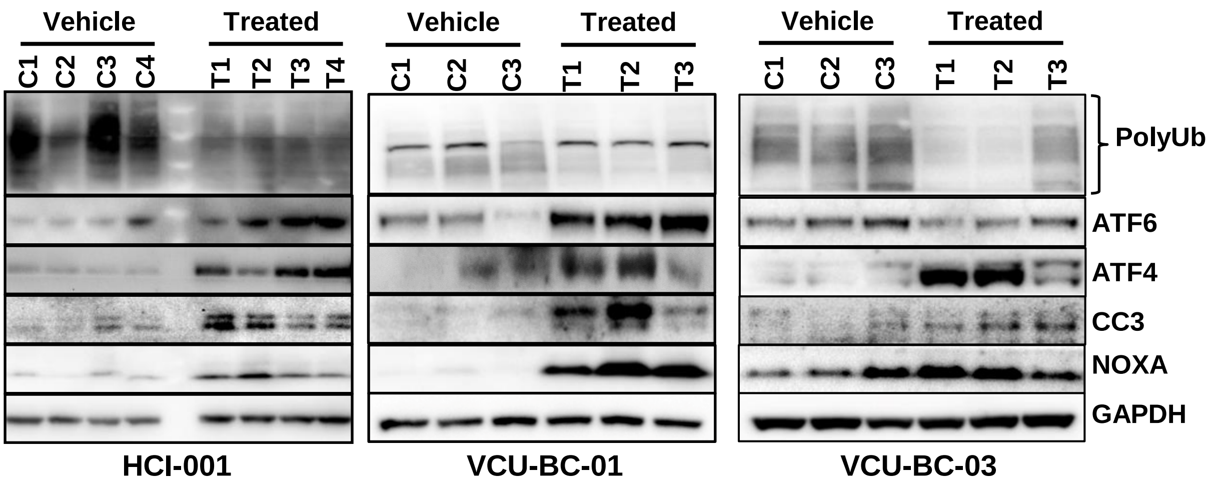

B

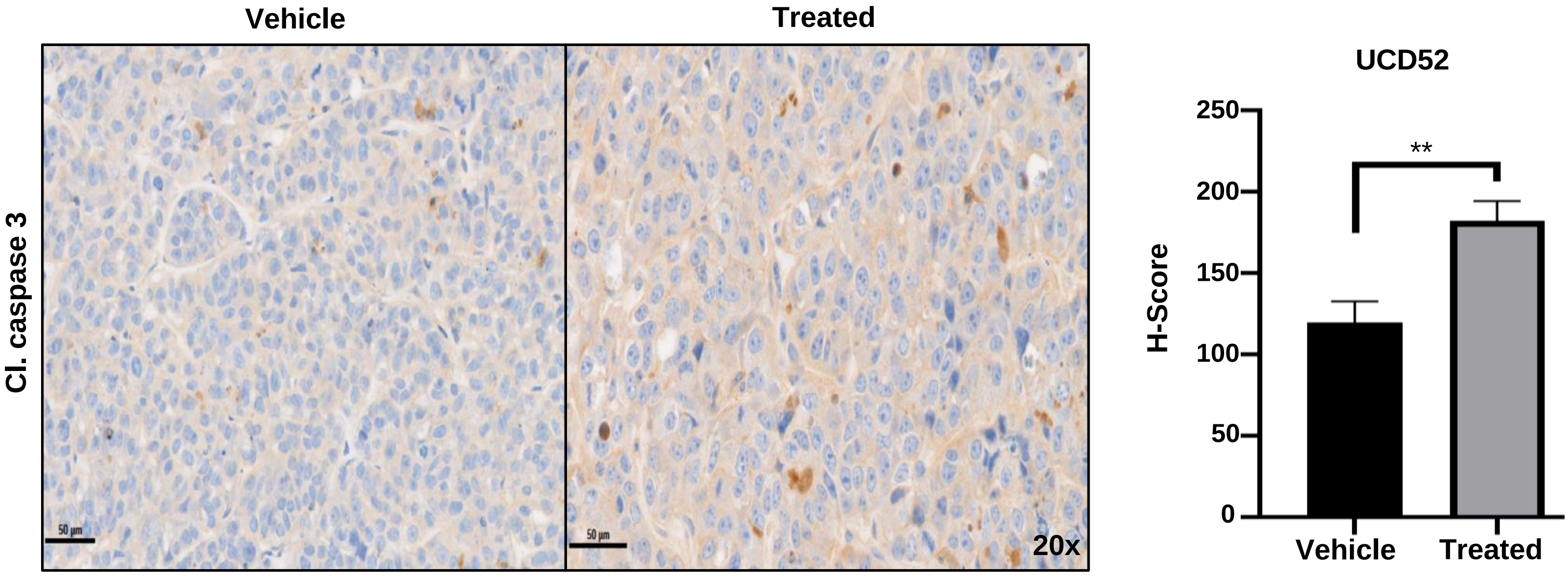

Figure S3. TAK-243 induces cell death *in vivo*. (A) Western blot analysis showing effects of TAK-243 on UPR and apoptotic proteins in PDX HCI-001, VCU-BC-01 and VCU-BC-03 mice treated with TAK-243, as assessed by immunoblotting for polyubiquitin (polyUb), ATF4, ATF6, CC3 and NOXA. GAPDH was used as a loading control. (B) Representative images of cl. caspase 3 (CC3) staining in UCD52 PDX of vehicle (DMSO) or TAK-243 (25mg/kg, biweekly for 3 weeks) treated tumors. Total magnification 20x (scale bar: 50µm). Graph indicates H-Score analysis of the staining in the vehicle and TAK-243 treated tumor tissues (**p<0.01).

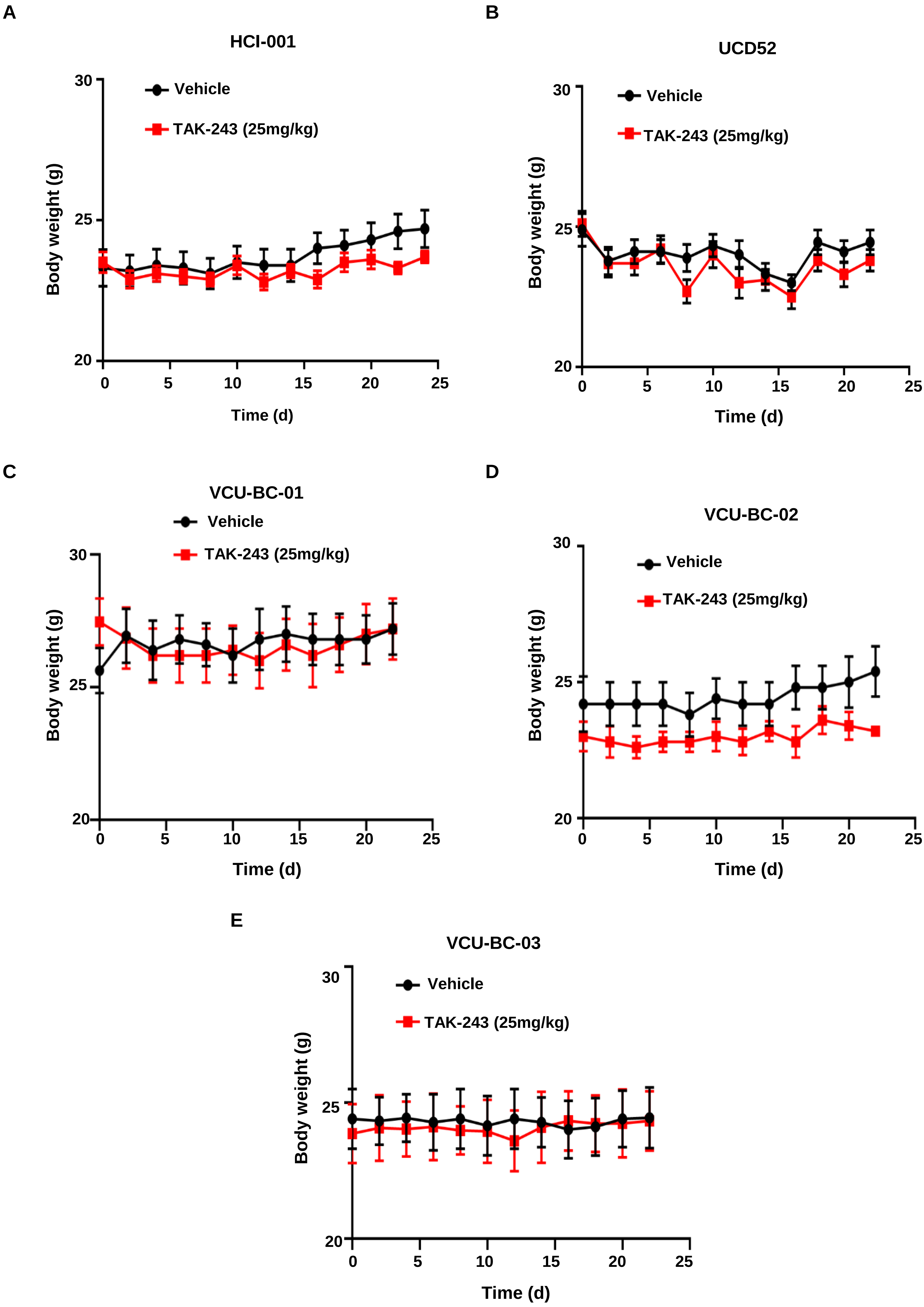

Fig S4. TAK-243 has minimal effect on animal weight. Graph displays the mouse weights of NSG mice bearing (A) PDX HCI-001, (B) PDX UCD52, (C) VCU-BC-01, (D) VCU-BC-02 and (E) VCU-BC-03 throughout the efficacy studies.

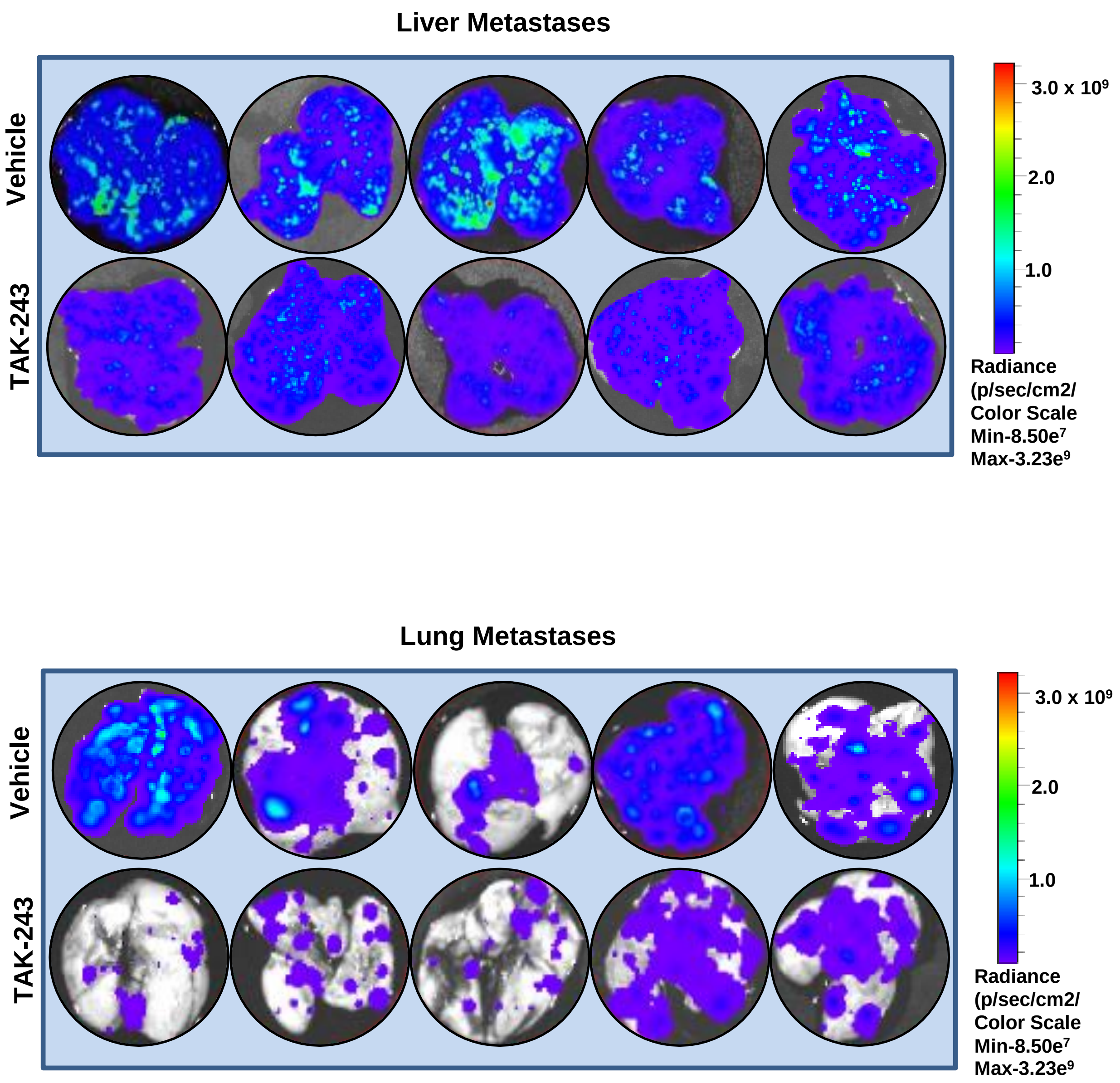

Fig S5. **TAK-243 decreases metastases in primary organs.** Images represent total metastases quantified *ex vivo* in the liver and lung of the vehicle and TAK-243 treated mice.
